# Imaging intact human organs with local resolution of cellular structures using hierarchical phase-contrast tomography

**DOI:** 10.1038/s41592-021-01317-x

**Published:** 2021-11-04

**Authors:** C. L. Walsh, P. Tafforeau, W. L. Wagner, D. J. Jafree, A. Bellier, C. Werlein, M. P. Kühnel, E. Boller, S. Walker-Samuel, J. L. Robertus, D. A. Long, J. Jacob, S. Marussi, E. Brown, N. Holroyd, D. D. Jonigk, M. Ackermann, P. D. Lee

**Affiliations:** 1 Department of Mechanical Engineering, University College London, London, UK; 2 Centre for Advanced Biomedical Imaging, University College London, London, UK; 3 European Synchrotron Radiation Facility, Grenoble, France; 4 Department of Diagnostic and Interventional Radiology, University Hospital Heidelberg, Heidelberg, Germany; 5 German Lung Research Centre (DZL), Translational Lung Research Centre Heidelberg (TLRC), Heidelberg, Germany; 6 Developmental Biology and Cancer Programme, Great Ormond Street Institute of Child Health, University College London, London, UK; 7 UCL MB/PhD Programme, Faculty of Medical Sciences, University College London, London, UK; 8 French Alps Laboratory of Anatomy (LADAF), Grenoble Alpes University, Grenoble, France; 9 Institute of Pathology, Hannover Medical School, Hannover, Germany; 10 German Center for Lung Research (DZL), Biomedical Research in Endstage and Obstructive Lung Disease Hannover (BREATH), Hannover, Germany; 11 Department of Histopathology, Royal Brompton and Harefield NHS Foundation Trust, London, UK; 12 National Heart and Lung Institute, Imperial College London, London, UK; 13 Centre for Medical Image Computing, University College London, London, UK; 14 UCL Respiratory, University College London, London, UK; 15 Institute of Functional and Clinical Anatomy, University Medical Center of the Johannes Gutenberg University Mainz, Mainz, Germany; 16 Institute of Pathology and Department of Molecular Pathology, Helios University Clinic Wuppertal, University of Witten-Herdecke, Wuppertal, Germany

**Keywords:** X-ray tomography, Translational research, Kidney, Cardiovascular biology

## Abstract

Imaging intact human organs from the organ to the cellular scale in three dimensions is a goal of biomedical imaging. To meet this challenge, we developed hierarchical phase-contrast tomography (HiP-CT), an X-ray phase propagation technique using the European Synchrotron Radiation Facility (ESRF)’s Extremely Brilliant Source (EBS). The spatial coherence of the ESRF-EBS combined with our beamline equipment, sample preparation and scanning developments enabled us to perform non-destructive, three-dimensional (3D) scans with hierarchically increasing resolution at any location in whole human organs. We applied HiP-CT to image five intact human organ types: brain, lung, heart, kidney and spleen. HiP-CT provided a structural overview of each whole organ followed by multiple higher-resolution volumes of interest, capturing organotypic functional units and certain individual specialized cells within intact human organs. We demonstrate the potential applications of HiP-CT through quantification and morphometry of glomeruli in an intact human kidney and identification of regional changes in the tissue architecture in a lung from a deceased donor with coronavirus disease 2019 (COVID-19).

## Abstract (web summary)

Hierarchical phase-contrast tomography (HiP-CT) enables multiscale imaging of any region within an intact human organ down to cellular resolution. HiP-CT of five organ types revealed 3D morphological features in healthy and diseased tissue.

## Main

Biological tissues are complex 3D structures arranged hierarchically from individual specialized cells to organotypic functional units, for example, alveoli in the lung up to intact, whole organs. Spatial relationships, 3D morphology and interaction within and across these length scales collectively provide a basis for biological function. Thus, mapping the spatial organization and morphology of individual cells up to the scale of intact organs is fundamental to understanding system-level behaviors in health or disease.

Spatial mapping at the single-cell level for entire human organs is presently unfeasible in terms of existing techniques, data-storage requirements and analysis or interpretability^1^. A more practical approach is to provide overall spatial context at a lower resolution and then use this data to select smaller regions for higher-resolution imaging; this type of imaging can be considered hierarchical imaging. Currently, hierarchical imaging typically involves physical subsampling of larger samples before high-resolution imaging^2^. Physical subsampling creates challenges in data registration and the requirement that correct or representative subsamples are collected^2,3^. New 3D imaging techniques are therefore required to bridge length scales from spatial relationships at the cellular level to the architectural organization of intact human organs.

In recent years, there has been progress toward achieving 3D imaging of intact organs at multiple length scales. One approach is optical clearing, which homogenizes the refractive index in biological tissues, allowing high-resolution 3D imaging modalities such as light-sheet microscopy or optical projection tomography. These techniques have allowed the visualization of cellular structures in human embryonic development^4,5^ and mouse models of cancer metastasis^6^ and drug uptake^7^. Optical clearing of whole adult human organs has recently been achieved; however, it requires several months for intact organs and causes changes in tissue morphology, and light-sheet microscopes cannot presently image whole organs in their intact state^8^. High-resolution magnetic resonance imaging (MRI) requires minimal tissue preparation, does not cause tissue distortion and achieves 100 μm per voxel in an ex vivo human brain^9^. However, this type of MRI scan requires 100 h for the human brain and does not achieve the resolution required to capture cellular detail. Multibeam electron microscopy can provide images of human tissue from cellular to subcellular scales^2^ but cannot capture the large volumes of tissue required for whole human organs.

Synchrotron X-ray tomography (sCT) is a promising approach to image whole human organs in detail at the cellular level^10,11^. X-rays are intrinsically suited to imaging different length scales owing to their deep penetration and short wavelength. Some specialized synchrotron tomography instruments have achieved high resolution relative to the overall size of the biological sample, including resolving cellular features in human spinal cord biopsies 32 mm in diameter^12^, achieving a resolution of ~5 µm in human lung biopsies (diameter of 6 mm)^13^ and a resolution of 87 nm in volumes of interest (VOI) within an intact *Drosophila* leg ~1 mm in diameter^14^. In these cases, the entire sample was scanned at the reported resolutions rather than a hierarchical series of resolutions performed with a single instrument. Local tomography or zoom tomography is an established synchrotron technique that enables this hierarchical approach. While it has been developed for use in small (500-μm) biological tissue samples, contrast agents such as barium or osmium are often used^1,15,16^. For large objects for which staining is far more challenging, local tomography has been confined to objects with high density differences, such as those found in bones or fossilized remains^17–19^. There are no established sCT techniques that achieve cellular resolution far from the central axis of rotation in large soft tissues such as human organs. Phase-contrast-based sCT^18,20,21^, specifically, propagation-based imaging^22^, can theoretically be applied to image large, soft tissues. However, this requires a high-energy X-ray beam to penetrate large samples, coupled with the high coherence and flux required to resolve cellular detail (and a beam size large enough to scan whole organs in reasonable time frames), which was previously not available in any laboratory source or at a single synchrotron beamline worldwide.

Recently, the first high-energy (6-GeV), fourth-generation, synchrotron source at the ESRF, named the EBS, has provided the beam coherence required to resolve faint density contrasts at high resolution while achieving a 100-fold increase in brilliance compared to its predecessors^23^. We have leveraged the latest iteration of the ESRF-EBS, using the test beamline BM05, to develop a technique termed HiP-CT. We demonstrate the utility of HiP-CT to image intact human tissues across length scales, spanning whole organs down to some specialized cells in VOI. We apply HiP-CT to image a series of intact human organs and provide quantitative and morphometric insights into the healthy human kidney and lung. We additionally apply HiP-CT to a contemporary biomedical problem by characterizing changes in the lung architecture of a patient with fatal COVID-19.

## Results

### HiP-CT development and implementation

**Fig. 1:**
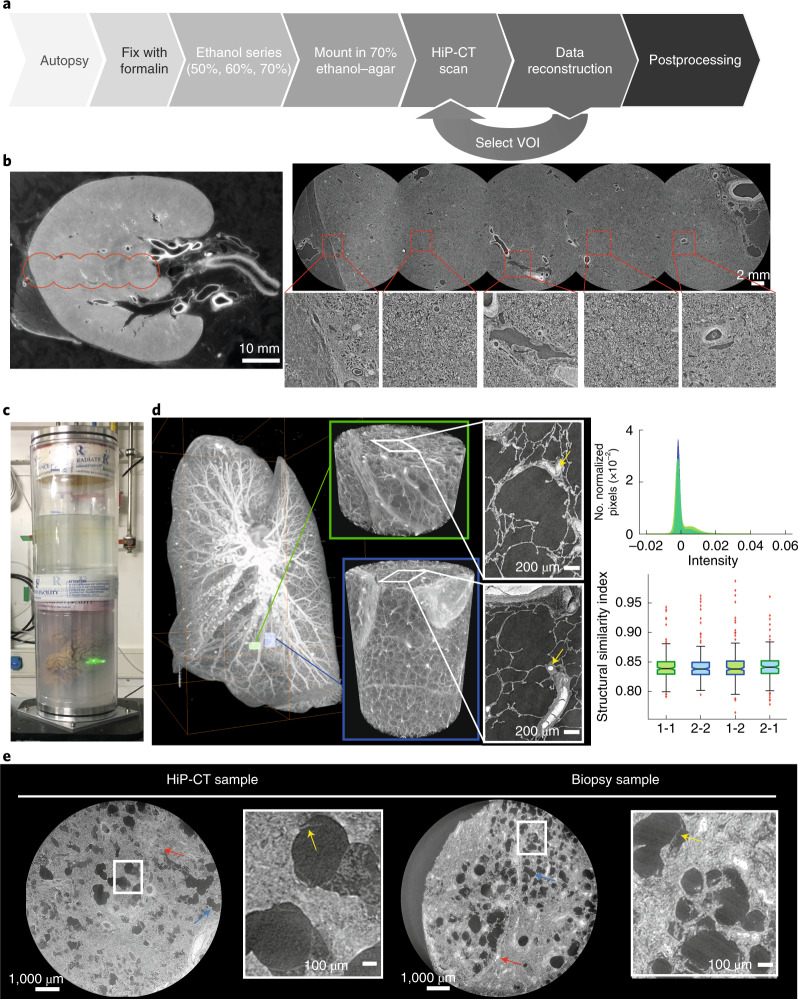
A HiP-CT pipeline for multiscale 3D imaging from whole-organ to cellular resolution within large intact soft tissue samples. **a**, Flow chart of HiP-CT sample preparation and imaging procedure; the ability to select specific higher-resolution scan regions based on lower-resolution scans provides hierarchical tissue structure images in a data-efficient manner. **b**, Left, 2D image slice (25 µm per voxel) showing the location of a series of regions of 2.5 µm per voxel that transect the organ’s radius (red circles). Right, HiP-CT scans at 2.5 µm per voxel every 7 mm from the external kidney surface (left) to the center of the sample (right). Scans are overlapped and stitched to provide a complete organ. The magnified view shows a constant level of data quality and precision over the complete transect through the use of the reference scan procedure. **c**, Photograph of an intact human brain mounted in a polyethylene terephthalate jar with ethanol–agar stabilization and with the reference jar on top. **d**, Left, maximum intensity projection of a whole human lung with two randomly selected VOI imaged at a resolution of 2.45 µm per voxel shown in green (VOI1) and blue (VOI2). Three-dimensional reconstructions of the two high-resolution VOI are shown with 2D slices in the insets. In the 3D high-resolution VOI, the fine mesh of pulmonary blood vessels and the complex network of pulmonary alveoli and their septa can be seen. Yellow arrows denote occluded capillaries in 2D slices. Top right, image stack histograms for the green (VOI1) and blue (VOI2) high-resolution VOI, respectively (fixed bin width, 0.0001). Intensity distributions are comparable with positive skew (1.82 and 2.68) and kurtosis (6.44 and 11.88) for VOI1 and VOI2, respectively; the histogram intersection is 71 ± 3% for fixed bin width in the range 1 × 10^−2^ – 3 × 10^−4^. Bottom right, box-and-whisker plot showing the structural similarity index between *n* = 200 pairs of 2D slices independently sampled either from within the same VOI (1-1 and 2-2) or from different VOI (1-2 and 2-1) for each group, respectively; one-way ANOVA (two sided); *P* = 0.8765, three degrees of freedom, *F* = 0.23). Box plots show the median (center line), interquartile range (75th–25th percentiles) of data (box bounds) and data range excluding outliers (whiskers)); values more than 1.5 times the interquartile range above or below box bounds are denoted as outliers (red crosses). **e**, Single representative slices of high-resolution scans from a HiP-CT image of an intact whole human lung lobe affected by COVID-19 (donor 3) and a biopsy taken from the same patient’s contralateral lung. Both VOI are captured from the upper peripheral region of each upper lung lobe. In HiP-CT images, fine structure of the tissue including blood capillaries (red arrows) and alveoli (blue arrows) as well as thin alveolar septa (yellow arrows in insets) is depicted. Source data

**Extended Data Fig. 1:**
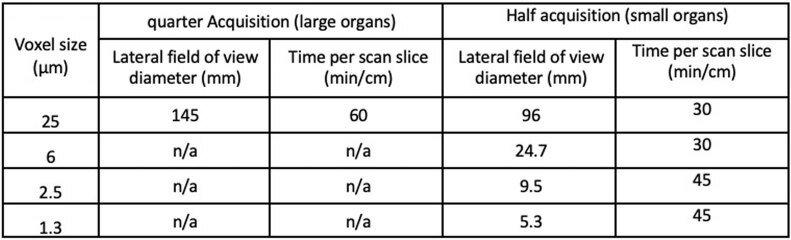
Minimum scan times. Minimum scan times for quarter and half acquisition modes at voxel sizes presented in this work.

HiP-CT requires the specific sample-preparation, scanning and reconstruction approaches shown in Fig. 1a (see Methods for detailed steps). Briefly, organs are fixed, partially dehydrated and physically stabilized for scanning (Fig. 1c). HiP-CT scans are then performed hierarchically, the first typically at 25 µm per voxel over the whole organ followed by VOI at 6.5 µm per voxel and 1.3–2.5 µm per voxel. Scanning time for HiP-CT is faster than that for other techniques^8,9^, currently ~16 h for a whole brain at 25 µm per voxel and ~3.5 h for a whole kidney at 25 µm per voxel (see Methods and Extended Data Fig. 1 for further details). Our experimental setup is largely automated and can be adjusted to increase scanning speed for smaller organs while accommodating different fields of view for higher-resolution imaging. Our scanning procedure allows users to select high-resolution VOI with the spatial context provided by the preceding lower-resolution scans (Supplementary Information).

Long-term physical stabilization of organs was necessary for HiP-CT, both to enable multi-resolution registration and to allow organs to be removed from the beamline while higher-resolution VOI were selected. Our optimized organ-stabilization method (see Methods for further details) using agar–agar gels was highly effective for maintaining organ positions (see Supplementary Video 1 in which the higher-resolution scans were taken several months after the lower-resolution scans and simply registered by manual rigid transformation) and uses easily obtained materials. Several other key procedures and modifications of the BM05 experimental hutch, summarized and prioritized in Table 1 and outlined in the Methods, describe essential developments for HiP-CT. Central to HiP-CT is the increased spatial coherence provided by the EBS. This spatial coherence allows propagation distances approaching the near-field limit for scans of the highest resolution and thus resolves faint density contrasts in soft tissue^1^.

**Table 1:** Overview of techniques and capabilities developed and used to enable HiP-CT

Imaging pipeline feature	Development and optimization		
Source and beam	E, fourth-generation high-energy storage ring with decreased source size and increased spatial coherence		I, polychromatic beam for simplified phase-contrast approach and high flux
Filters, optics	**E, use of high-purity and highly polished windows and filters to preserve spatial coherence**		I, metallic filters and fused silica bars to allow tuning of flux, beam profile and energy
Optimized scan parameters	**NC, fine tuning of beam properties to balance transmission through sample against scan speed**		**E, longest possible propagation distance until the limit of geometric blurring (or physical hutch limit)**
Scanning geometry	**E, exploiting the horizontal beam profile to adapt to the attenuation profile of the sample at low resolution**		**E, performing scans in an equivalent jar with only mounting medium to generate adaptive flat-field references to normalize the absorption profile**
Scintillator	**I, using a high-light-output, high-atomic number, high-density crystal scintillator for optimal quality at high energy (LuAG:Ce)**		**NC, optimizing scintillator thickness for each magnification to find the best compromise between pure resolution and signal level**
Detector optics	**NC, using optics with the highest possible numerical aperture to optimize light-collection efficiency. This was matched to the scintillator thickness.**		**NC, making detector radiation hard to limit the darkening of the optics by use of lead glass and glassy carbon mirrors. Rapid restoration process for darkened detectors using blue LEDs**
Detector sensors	**NC, 2,048** **×** **2,048-pixel sCMOS with small pixels used to ensure rapid readout, high quantum efficiency and low electronic noise**		I, recovery of large full-well capacity through the use of frame accumulation when required (at lower resolution when attenuation contrast is dominant)

Cells in bold were specifically developed or optimized for HiP-CT. E, essential; I, important; NC, non-critical.

LED, light-emitting diode; sCMOS, scientific complementary metal oxide semiconductor.

Much of the HiP-CT scanning geometry aims to reduce X-ray dose to the sample, optimize the dynamic range of the detector, reduce ring or bubble artifacts and suppress beam hardening. Matching the horizontal Gaussian profile of the beam to the attenuation profile of the sample at 25 μm per voxel achieves all of the above benefits (see Methods for details). The dose limit is crucial in HiP-CT, because, once it is surpassed, bubbling is induced in the sample, causing tissue damage and introducing artifacts (see Supplementary Information for further details).

**Extended Data Fig. 2:**
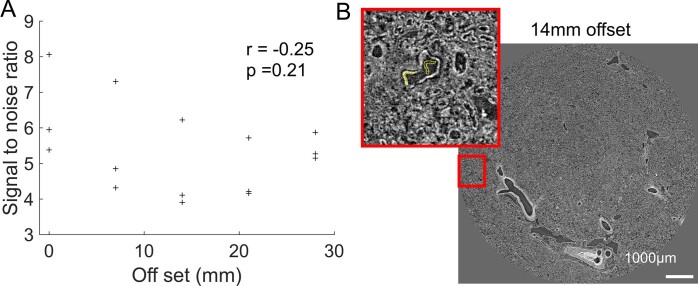
Signal to noise variation with lateral distance from center of rotation. Graph showing the signal to noise variation with lateral off-set from centre of rotation. Pearson correlation coefficient (r) = -0.25 and p-value =0.21 (two-tailed test) shown on graph. It should be noted that the exposure time of the camera and final selection of the X-ray filters were set-up on the most off-centre scan, at the angle where the beam was crossing the smallest amount of material, in order to ensure that detector saturation would not occur during scanning. B) showing an example of the regions used to calculate the SNR. Signal was taken from tubule walls while the open tubule lumen was used as the background region.

To perform extreme off-axis local tomography reconstructions, we have developed the use of hierarchical reference scans. For every higher-resolution VOI, a reference scan is taken in an identical jar filled with an agar–ethanol mixture mounted on top of the sample jar (Fig. 1c). This scan provides a background estimate that can be removed when performing reconstruction, thus eliminating much of low-frequency background variations. To demonstrate the effectiveness of the reference scan method, we performed a series of high-resolution scans transecting the kidney from the center of rotation to the outer edge (Fig. 1b, left). The consistent image quality across the high-resolution scans (Fig. 1b, right) can be appreciated in the magnified insets and the lack of correlation between the signal-to-noise ratio (SNR) and lateral scan position (Pearson’s correlation coefficient, −0.25; *P* = 0.21; Extended Data Fig. 2).

The HiP-CT scanning procedure adapts two protocols originally developed to image large fossils. The first, the attenuation protocol, normalizes absorption in the field of view^24,25^; whereas the second, the accumulation protocol, provides extended dynamic range^26^. At 25 μm per voxel, HiP-CT scans are dominated by attenuation; hence, there is reduced contrast sensitivity and a requirement for more accumulations, whereas higher resolutions are dominated by phase, leading to fewer accumulations (see Table 2 and Supplementary Data 1 for all scanning parameters). The dominance of attenuation at lower resolution creates horizontal line artifacts during concatenation of radiographs due to the vertical profile of the beam. To remove these artifacts, the vertical profile residual background after flat-field correction is subtracted before reconstruction. For higher-resolution scans in which phase effects dominate, this step is not required (see Extended Data Fig. 6 and Methods for details).

**Table 2:** Scan parameters for all samples

Sample	Voxel size (µm)	Data label	Average energy of the incoming beam (keV)	Lateral field of view (diameter (mm))	Scan time (h)
Donor 1, heart	25.08	Complete organ	~85	96	18
	6.05	Left and right ventricle muscle with ramus interventricularis anterior	~87	24.7	6
	2.22	Left ventricle muscle	~76	9.5	4
Donor 1, left lung	25.08	Complete organ	~85	145	24
	25.25	FSC A and B	~80	145	3
	6.05	VOI-06	~81	23	4
	6.5	FSC A and B	~80	24.7	2
	2.45	VOI-02	~70	9.5	3
	2.45	VOI-06	~70	9.5	3
	2.51	FSC A and B	~70	9.6	2
Donor 1, left kidney	25.08	Complete organ	~85	85	2
	6.05	Central column	~85	24.7	2
	1.29	Central column	~69	5.3	3
Donor 1, spleen	25.08	Complete organ	~85	85	2
	6.05	Central column	~85	24.7	1
	1.29	Central column	~69	5.4	2
Donor 2, brain	25.08	Complete organ	~85	145	22
	6.05	Cerebellum, occipital lobe	~81	24.7	5
	2.45	Cerebellum	~74	9.5	5
Donor 2, kidney	25	Complete organ	~84	85	5
	2.5	Lateral transect	~80	9.5	1
Donor 3, upper lobe of left lung	26.38	Complete upper lobe	~66	96	11
	6.24	VOI-4		24.7	24
	2.22	VOI-1.2b	~64	24.7	16
	2.22	VOI-8.2	~76	9.5	7
Donor 3, upper lobe of right lung	2.25	Core biopsy	~43	9.5	3
Donor 4, lower lobe of right lung	2.25	Core biopsy	~78	9.5	2

After preprocessing of radiographs to generate high-quality local tomography (see Methods for further details), image reconstruction can be performed using a filtered back-projection algorithm, coupled with single-distance phase retrieval^27^, combined with a two-dimensional (2D) unsharp mask performed on the projection phase maps as implemented in PyHST2 software^28^. All subscans (covering typically 2.5 mm vertically) are concatenated after reconstruction. Subsequently, residual ring artifacts that would not have been removed in the preceding steps are corrected on reconstructed slices^29^.

To assess the performance of HiP-CT for imaging human organs, we scanned the intact human lung of a 94-year-old female (donor 1, clinical details are in the Methods), beginning with the whole organ at 25 µm per voxel, before selecting VOI for magnified imaging at 6.5 µm per voxel and 2.5 µm per voxel (Fig. 1d).

**Extended Data Fig. 3:**
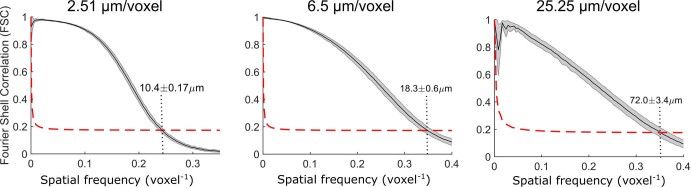
Resolution estimation via Fourier shell correlation. Resolution estimation from fourier shell correlation (FSC) measure. FSC curves (black) with standard deviation (grey shaded) and ½ bit criterion (red hatched) for each voxel size imaged Donor 1 lung. Intercept point is marked and labelled with the equivalent spatial resolution. FSC was calculated in each case using 9 subvolumes cropped at randomly varying spatial locations from the total image volume. Subvolume sizes were varied (200,500 and 1000 voxels).

We estimated the resolution of HiP-CT by Fourier shell correlation (FSC) analysis^1,16^. Resolution was estimated at the half-bit criterion to be 10.4 µm ± 0.17 µm for images at 2.5 µm per voxel, 18.3 µm ± 0.6 µm for images at 6.5 µm per voxel and 72 µm ± 3.4 µm for images at 25 µm per voxel (Extended Data Fig. 3). To assess the consistency in the quality of higher-resolution scans at different tissue depths and distances from the rotational center of the intact organ, we analyzed intensity distributions across two high-resolution VOI. We found that the image intensity histograms had an intersection of 71% ± 3%. Both distributions showed positive skewing and kurtosis with minimal differences in mean intensities between the different VOI (Fig. 1d, top right). In addition, we quantified differences in image quality between the two volumes using the structural similarity index^30^ (Fig. 1c and Fig. 1d, bottom right). No significant difference in structural similarity index (median values, 0.839–0.841, *P* = 0.88) was observed across compared image volumes, indicating that HiP-CT can achieve high-resolution scanning in any region of the intact human lung with consistent quality.

The large size of adult human organs is a fundamental imaging challenge: the penetration depth of imaging photons (X ray for HiP-CT) and diffusion of reagents (ethanol for HiP-CT) may both be limiting factors. Accordingly, image quality tends to decrease from small to large specimens. We therefore qualitatively compared high-magnification HiP-CT scans of an intact human lung and a physically subsampled biopsy (cylindrical biopsy, 8.1 mm in diameter, 14 mm in height). The image quality of the two samples was found to be indistinguishable (Fig. 1e). The fine structure of the lung tissue including capillaries and alveoli was depicted in a histopathologically correct and undistorted manner, with very thin membranes within the alveoli (yellow arrows) being some of the smallest structures (thickness of ~5 µm) evident in both samples.

Thus, HiP-CT is versatile, providing high-quality imaging of human lungs across multiple length scales, independent of the region sampled or the size of the tissue imaged.

### HiP-CT imaging of whole human organs

**Fig. 2:**
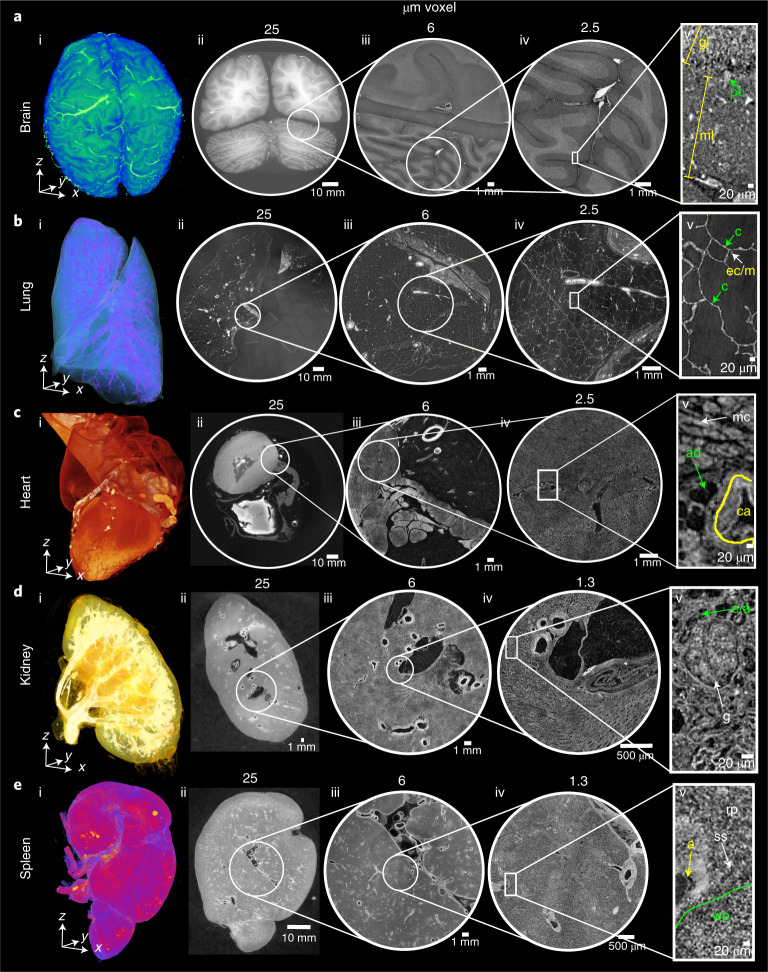
HiP-CT enables 3D imaging of organotypic functional units across intact human organs. HiP-CT of brain (**a**), lung (**b**), heart (**c**) kidney (**d**) and spleen (**e**); for each organ, 3D rendering (i) of the whole organ is shown using scans at 25 µm per voxel. Subsequent 2D slices (ii–iv) show positions of the higher-resolution VOI relative to the previous scan. (v), Digital magnification of the highest-resolution image with annotations depicting characteristic structural features in the brain (ml, molecular layer; gl, granule cell layer; pc, Purkinje cell), in the lung (c, blood capillary; ec/m, epithelial cell or macrophage), in the heart (mc, myocardium; ca, coronary artery; ad, adipose tissue), in the kidney (e/a, efferent or afferent arteriole; g, glomerulus) and in the spleen (rp, red pulp; wp, white pulp; a, arteriole; ss, splenic sinus). All images are shown using 2× binning.

Next, we sought to apply HiP-CT to image multiple length scales in a variety of human organs. Intact lung, heart, kidney and spleen (donor 1) and brain (donor 2), were acquired from two donors (clinical details in the Methods), processed and imaged by HiP-CT. Images were sequentially acquired at 25 µm per voxel to capture the entirety of each organ, before scans at 6 µm per voxel and 2.5 or 1.3 µm per voxel of selected VOI (Supplementary Videos 1–4). At 25 µm per voxel, macroscopic features of each organ were unambiguously identifiable through anatomical location and morphology including sulci and gyri of the cerebral cortex (Fig. 2a(i,ii) and Supplementary Videos 1 and 4), individual lobules of the lung (Fig. 2b(i,ii) and Supplementary Videos 2 and 4), the four chambers of the heart and associated coronary arteries (Fig. 2c(i,ii) and Supplementary Video 4), the pelvis and calyces of the kidney (Fig. 2d(i,ii) and Supplementary Videos 3 and 4) and the pulpa of the spleen (Fig. 2e(i,ii) and Supplementary Video 4).

**Extended Data Fig. 4:**
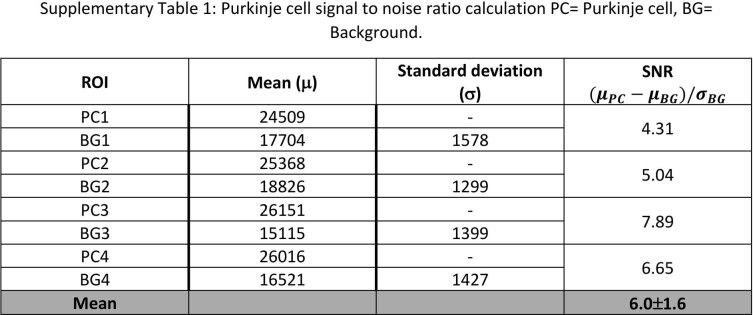
Purkinje cell signal to noise ratio. Purkinje cell signal to noise ratio calculation PC= Purkinje cell, BG= Background.

Human organ function is driven by the collective activity of organotypic functional units. Each functional unit comprises multiple spatially arranged cell types that facilitate specialized functions. We used high-resolution HiP-CT to image functional units across the same five human organs (Fig. 2). The hierarchical nature of HiP-CT, isotropy of the image data and high resolution facilitated identification of specific organotypic structures and, in certain cases, specialized cells. The use of comparative and correlative histology is a further aid for distinguishing specific structures and cell types (Supplementary Video 4). In the brain, layers of the cerebellum were visible (Fig. 2a(iii,iv)), and a number of individual Purkinje cells were evident between the molecular and granule cell layers, identifiable by their distinctive pyramidal cell body and anatomical location^31^ (Fig. 2a(iv,v) and Supplementary Video 4). The SNR of four Purkinje cells was 6 ± 1.6 (Extended Data Fig. 4). In the lung, the intralobular septa and septal veins were visible (Fig. 2b(iii)) along with the terminal bronchi, which lead into acini (Supplementary Videos 2 and 4). Within the acini, cup-shaped alveoli, across which gas exchange occurs, brightly contrasted cell-sized objects mainly at intersections of alveolar septa, were evident, which we identified as type II pneumocytes and/or alveolar macrophages based on comparative histology (Fig. 2b(iv,v) and Supplementary Video 4). Bundles of cardiac muscle fibers were visible in the heart (Fig. 2c(iii)), consisting of individual cardiomyocytes, which could be distinguished by their distinctive shape and arrangement in fascicles and by comparative histology^32^ (Fig. 2c(iv,v) and Supplementary Video 4). In the kidney, epithelial tubules comprising the nephron were evident (Fig. 2d(iii)), which, at their apex, harbored the intricate capillary network of the glomerulus (Fig. 2d(iv,v) and Supplementary Video 4), specialized for filtration of blood. Finally, the organization of red and white pulp in the spleen (Fig. 2e(iii)) was visible, the former containing splenic sinuses and the latter containing periarterial lymphoid sheaths and lymphocyte-rich follicles (Fig. 2e(iv,v) and Supplementary Video 4). Collectively, these images show that HiP-CT is capable of imaging intact human organs down to the resolution of organotypic functional units and certain types of specialized cells.

### Quantitative HiP-CT in the healthy human kidney

Next, we sought to assess the utility of HiP-CT to obtain physiologically relevant structural information, using the human kidney as an example. The functional capacity of the human kidney arises from the collective activity of individual units called nephrons. Each nephron has a glomerulus: a network of blood capillaries that is the site of blood filtration. The total number of glomeruli (*N*_glom_) is indicative of the kidney’s capacity for filtration, and, as nephrons are not (re)generated during the life of an individual^33^, reduction in *N*_glom_ is a feature of physiological aging and chronic kidney disease^34–36^.

**Fig. 3:**
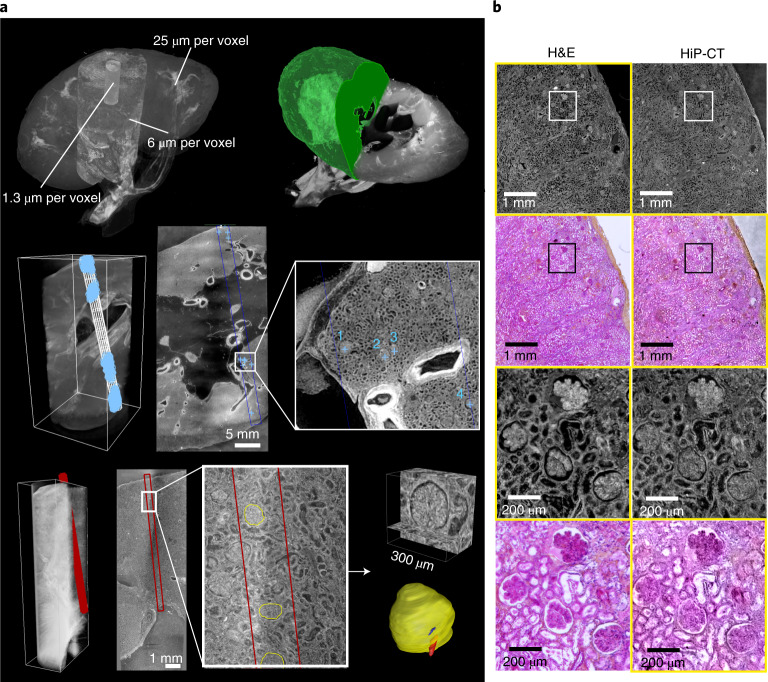
HiP-CT analysis of the kidney to measure glomerular morphology and nephron number. **a**, Top left, HiP-CT datasets at three resolutions (25, 6 and 1.3 µm per voxel) obtained from a human kidney, aligned and overlaid. **a**, Top right, measurement of the parenchymal volume, semi-automatically segmented (green). **a**, Middle, the dataset at 6 µm per voxel with the virtual biopsy cylinder is shown in white; the parenchymal volume within the cylinder was measured. A representative 2D slice with an inset shows four labeled glomeruli. In total, 853 glomeruli that were within the cylinder were counted; a blue + sign is used to denote the approximate center of each glomerulus. **a**, Bottom, the dataset at 1.3 µm with virtual biopsy (red cylinder). The 13 glomeruli within this cylinder were segmented in 3D as shown in the 2D representative slice with inset. **b**, Comparison of HiP-CT with an aligned histopathological section (*n* = 1) (stained with hematoxylin and eosin (H&E)) taken after all HiP-CT scanning was finished. The left-hand column shows light micrographs of H&E-stained histopathological sections, and the right-hand column shows 2D tomograms of HiP-CT; yellow boxes denote images that have been pseudocolored (from HiP-CT) or converted in gray levels and inverted in contrast (from histological observations). Source data

We assessed *N*_glom_ in a 94-year-old female (clinical information in the Methods (donor 1)) with HiP-CT (Supplementary Video 3 and Fig. 3a, top left). At 25 µm per voxel, the parenchymal volume was segmented (Fig. 3a, top right); at 6 µm per voxel, the number of glomeruli within a representative volume were counted (Fig. 3a, middle). *N*_glom_ was calculated as 310,000 for this individual, which is within the range of previous studies using either stereological analysis^34,36^, contrast-enhanced MRI^37^ or computed tomography^35^, and specifically agrees well with measures of *N*_glom_ in older individuals^36,38^.

**Extended Data Fig. 5:**
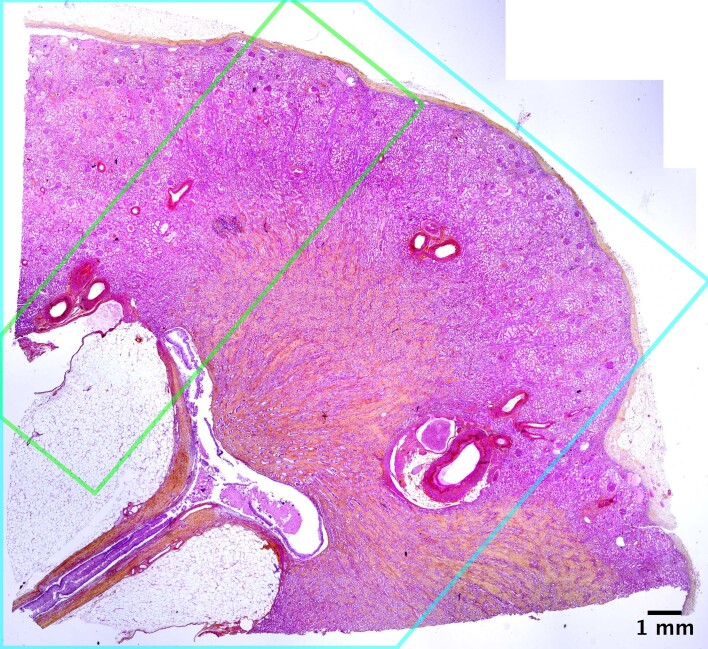
High X-ray dose does not cause detectable damage to kidney as assessed standard histology H&E. Large scale view of Haemotoxylin and Eosin (H&E) stained slice of the kidney with outlines of the 6 µm/voxel (blue) and 1.3 µm/voxel (green) scanning regions shown. There is no visible difference to suggest X-ray damage to parenchyma between these regions (N = 1).

Using scans at 1.3 µm per voxel, we also assessed glomerular volume, an important structural determinant of overall kidney health, which was shown to increase in individuals with reduced nephron number, obesity and hypertension^34,36^. Thirteen glomeruli were manually segmented to calculate *V*_glom_ (Fig. 3a, bottom), resulting in a value of 5.05 ± 0.09 × 10^−3^ mm^3^, similar to the range of *V*_glom_ (3–5 × 10^−3^ mm^3^) estimated by stereological and MRI analyses^36,37^. Additionally, the isotropy of HiP-CT enabled calculation of mean glomerular surface area (1.7 ± 0.4 × 10^5^ μm^2^) and sphericity (0.58 ± 0.09), both of which cannot be assessed using conventional modalities^38^. Moreover, we matched microscopy images of histological sections to the same regions of the kidney imaged with HiP-CT, finding equivalent structural detail in the two modalities (Fig. 3b). Histological results validate the accuracy of glomerular segmentation and show that sample preparation and exposure to high doses of X-rays during HiP-CT do not cause evident morphological tissue damage (Extended Data Fig. 5). Therefore, HiP-CT has the potential to quantify functional units and their 3D morphology with histological resolution within intact human kidneys, providing morphometric insights across entire organs.

### Quantitative HiP-CT characterization of the SARS-CoV-2-infected lung

We finally sought to apply HiP-CT to a contemporary biomedical problem by examining structural changes in the lungs of a patient diagnosed with COVID-19, caused by severe acute respiratory syndrome coronavirus 2 (SARS-CoV-2). The major cause of morbidity and mortality in COVID-19 is severe acute respiratory distress syndrome (ARDS)^39^. The clinical course of COVID-19 is well described^40^, and established histological features of SARS-CoV-2-infected lungs include alveolar inflammation, fibrosis and necrosis^41^. Recently, these findings were built upon by sCT and 3D reconstruction of millimeter-thick samples from COVID-19 lungs, demonstrating hyaline fibrotic deposits, lymphocytic infiltrates and vasculature occluded by thrombi^42^.

**Fig. 4:**
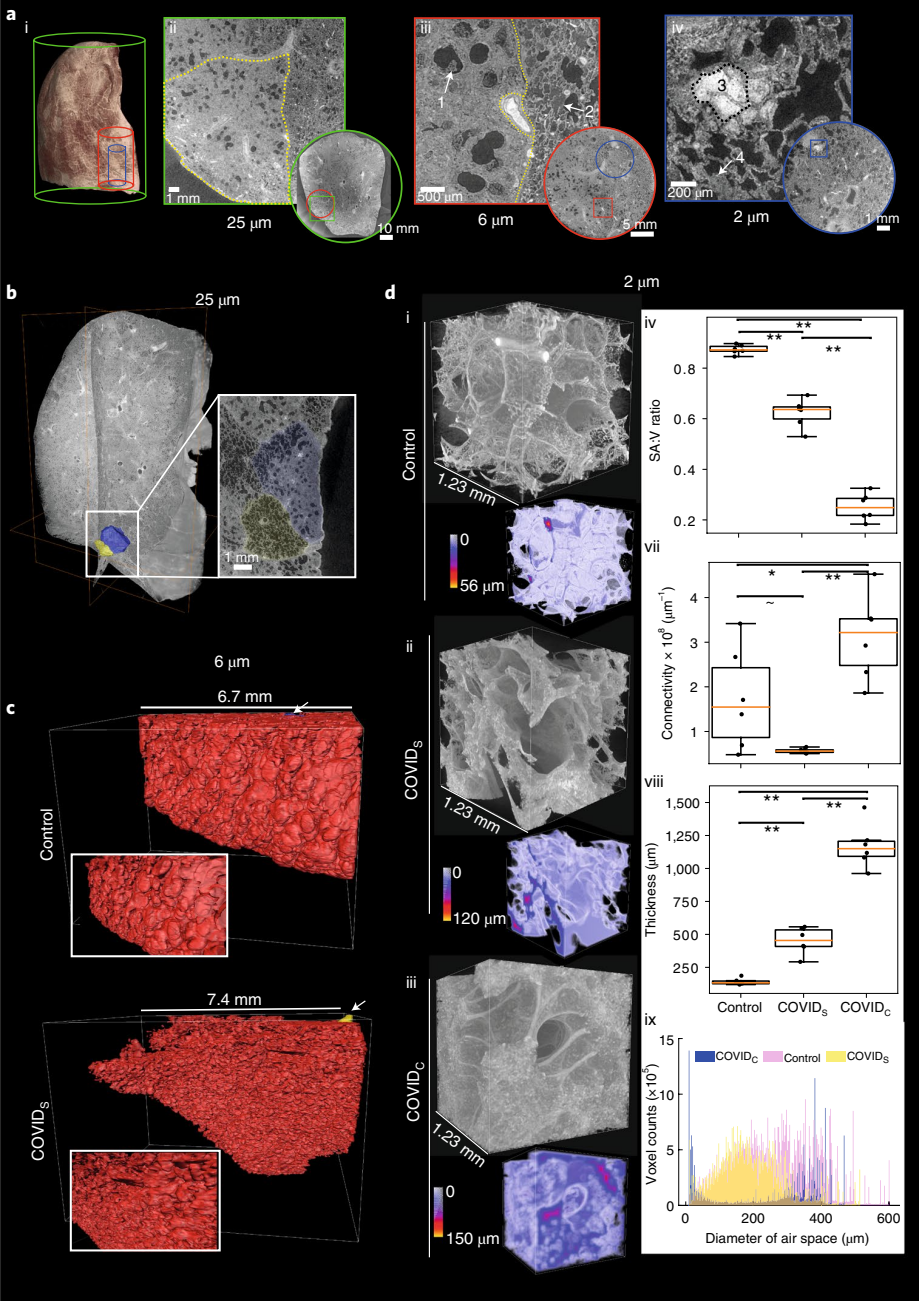
HiP-CT with 3D image analysis and morphometry in the lung of a patient with COVID-19. **a**(i), A 3D reconstruction from HiP-CT scanning at 25 µm per voxel of the intact upper left lung lobe from the autopsy of a patient who died from COVID-19-related ARDS. High-resolution VOI are shown in red (6.5 µm per voxel) and blue (2.5 µm per voxel). **a**(ii), At 25 µm per voxel, high-intensity regions are observed in the lung periphery. The yellow dotted line delineates a secondary pulmonary lobule. **a**(iii), At 6.5 µm per voxel, heterogeneity in the lung parenchyma included (1) dilated alveolar ducts and diffuse loss of alveolar structural organization and (2) comparatively well-preserved alveolar structure with some edematous changes. **a**(iv), At 2.5 µm per voxel, we observed (3) alveolar obstruction, likely representing clotted blood based on its high intensity and (4) interstitial thickening of alveolar septa. **b**, A 3D reconstruction of the COVID-19 lung with segmentation of two adjacent secondary pulmonary lobules with differing degrees of parenchymal deterioration. **c**, A 3D reconstruction of segmented acinus structure within the SARS-CoV-2-uninfected (control) lung (top) and the SARS-CoV-2-infected lung (bottom). **d**(i–iii), A 3D reconstruction of representative VOI at high resolution (2.5 µm per voxel) for the control, COVID_S_ and COVID_C_ groups, respectively. Duplicated volumes show visual representations of the airspace–tissue interface in the COVID-19 lung, where a smaller distance between a voxel of airspace and a voxel of tissue is colored blue, whereas larger distances are colored yellow. **d**(iv–viii), Box plots showing quantitative comparisons between *n* = 6 independent COVID_S_, COVID_C_ and control VOI. Box plots show data median (center line), the interquartile range (25th–75th percentiles) of data and data range without outliers (box bounds); outliers were considered as values 1.5 times above or below the box bounds (whiskers). Quantification of the mean surface area-to-volume (SA:V) ratio, airspace connectivity and mean septal thickness are shown, respectively (**P < 0.001, *P < 0.05 and ~*P* = 0.08) (calculated by two-tailed one-way ANOVA with Tukey’s comparison; *P* values and *F* statistics can be found in the Supplementary Information). **d**(ix), The distribution of airspace diameters for all six VOI in each group (modal values for COVID_C_, COVID_S_ and control, 8.9, 152 and 351 µm, respectively). Source data

To assess the utility of HiP-CT to detect changes in SARS-CoV-2-infected lungs, we imaged an intact upper right lung lobe acquired from the autopsy of a 54-year-old male patient who died from COVID-19-related ARDS (Fig. 4a). Clinical features of this patient are described in the Methods. Orthogonal 2D slices through the volume of the lung imaged at 25 μm per voxel demonstrated high-intensity regions in the lung periphery (Fig. 4a(ii) and Supplementary Video 5), consistent with patchy lung consolidation described by conventional clinical radiology in COVID-19^43^. Upon higher-resolution scanning of VOI at 6 μm per voxel, 2D slices depicted heterogeneity in the loss of normal alveolar architecture, with particular secondary pulmonary lobules displaying greater parenchymal deterioration than others (Fig. 4a(iii)). Higher magnification of the more affected secondary pulmonary lobule at 2 μm per voxel (Fig. 4a(vi)) demonstrated cavitation of lung parenchyma, alveolar obstruction (likely from thrombi, based on their contrast, similar to that of intravascular blood), thickening of septa between adjacent alveoli and blood capillary occlusion with adjacent cellular infiltrates (likely aggregations of lymphocytes^41^) (Supplementary Video 5). These results indicate that HiP-CT is capable of reproducing the microstructural findings observed in COVID-19 lung biopsies (Extended Data Fig. 7)^41,42^ across large tissue volumes and in providing access to structures at new length scales, for example, the secondary pulmonary lobule (Fig. 4b).

Given the heterogeneity of parenchymal damage, we aimed to characterize changes in lung architecture in differing regions of the lobe with varying levels of parenchymal deterioration. We identified and segmented two adjacent secondary pulmonary lobules from the periphery of the COVID-19 sample (Fig. 4b) that showed differing parenchymal deterioration, termed COVID_S_ (less deteriorated) and COVID_C_ (more deteriorated). For the COVID_S_ lobule (yellow), we used higher-resolution images at 6 µm to segment an acinus (Fig. 4c, bottom) and compared this to an acinus segmented from the control lung (Fig. 4c, top). These images show the loss of overall surface area in the lung and smaller and less uniformly shaped alveoli. To quantify this difference, we segmented the free air space using scans at 2.5 μm per voxel in VOI from COVID_C_ and COVID_S_ regions and compared these to those from an uninfected control lung (*n* = 6 VOI per group) (Fig. 4b). A 3D quantitative analysis was performed to assess microstructural changes, revealing a significant decrease in the surface area-to-volume ratio (Fig. 4d(vii)) and increase in septal thickness (Fig. 4d(viii)) between control versus COVID_S_, COVID_S_ versus COVID_C_ and control versus COVID_C_ regions (*P* < 0.001). Airspace connectivity was significantly higher for COVID_C_ than that for control regions (*P* = 0.03) and significantly higher than that for COVID_S_ regions (*P* < 0.001). These data (Fig. 4d(i–iii)) and the distribution of airway diameters (Fig. 4d(ix)) collectively quantify the heterogeneity of lung deterioration in SARS-CoV-2 infection. Thickening of alveolar septa leads to decreased airspace connectivity and a decrease in the modal airway diameter. In consolidated areas, infiltration of loose connective tissue and fluid into the alveoli leaves only tiny unconnected portions of the alveoli and the large well-connected alveolar ducts ventilated. This is evident in the bimodal distribution of airway sizes and high airspace connectivity. Thus, HiP-CT detected regional changes in the architecture and morphology of the COVID-19 lung and allowed quantification, which can inform our understanding of the pathogenesis of COVID-19-related ARDS in SARS-CoV-2 infection.

## Discussion

Here, we developed HiP-CT, a new phase-contrast-based sCT modality using the first high-energy fourth-generation synchrotron source^23^. Through our various methodological developments, we have enabled hierarchical 3D imaging of multiple intact human organs, providing consistently high imaging quality from whole human organs down to individual organotypic functional units and certain specialized cells at any location within the organ.

Due to mild sample preparation and non-destructive imaging, HiP-CT samples can be subsequently compared with those used in other imaging techniques, for example, histology, to provide validation and aid in HiP-CT image labeling. Our technique has several key advantages over previous sCT-based methods^1^: (1) images of different resolution from any location can be easily aligned with one another, facilitating data visualization and interpretation; (2) whether a high-resolution region is representative of the whole organ can be easily assessed; and (3) data collection and storage are efficient, particularly for rare and or spatially distant features. Our exemplar of multiscale glomerular analysis demonstrates the benefit of hierarchical imaging with representative subsampling to estimate the number and morphology of spatially disparate (glomerular) organotypic features. We also leveraged the advantages of HiP-CT for multiscale and quantitative 3D analyses in pathological contexts, identifying and quantifying regional changes in the architecture of the air–tissue interface and alveolar morphology in the lung of a donor with confirmed pulmonary COVID-19 disease.

Mapping the hierarchical structure of human organs is a major challenge in biology. International consortia to map the human body include the Human Cell Atlas and the Human Biomolecular Atlas Program^44,45^. Such consortia have generated data of the transcriptional states and cellular and molecular composition of human tissues^46^. To integrate HiP-CT into such efforts, a future challenge will be the validation and extension of automated cell detection and labeling. This may necessitate the development of contrast agents^47^ or of multimodal imaging using techniques with established targeted labeling^3^. Another challenge is the computational power and storage required to handle the magnitude of data generated by HiP-CT. While the hierarchical nature of HiP-CT is inherently data efficient, a single VOI through the depth of the lung captured at 6 μm per voxel still amasses ~600 GB of data. Emerging cloud-based frameworks^48^ could improve the speed of image processing and analysis and facilitate open accessibility of HiP-CT, providing a means to complement global efforts to map the human body.

HiP-CT has considerable translational potential for biomedical applications, which we demonstrated by 3D imaging of a SARS-CoV-2-infected lung. In addition to reproducing the histopathological hallmarks of COVID-19, HiP-CT revealed extensive regional heterogeneity in parenchymal damage. Our HiP-CT approach clearly demonstrates how physical subsampling methods that aim to infer organ-wide pathophysiology could be confounded by such heterogeneity. This potential confounder is surmounted by HiP-CT due to its flexibility in the scale of tissue volume that can be imaged, independent of location and over a wide range of resolutions. Moreover, quantitative analysis of lung architecture from HiP-CT images aligns with clinicopathological observations of an increased volume of ventilated air that does not participate in gas exchange in COVID-19-related ARDS^39,41^. Further HiP-CT investigation of COVID-19-related ARDS requires refinement using image-analysis methodologies such as machine learning^49^. HiP-CT could also be used to provide insights into the secondary consequences of COVID-19 in other organs, such as the kidney and brain, both of which show evidence of tropism for SARS-CoV-2^50^.

HiP-CT will evolve alongside advances in synchrotron technology. Here, we present results using our experimental method developed on a test beamline (BM05). The completion of a new beamline at the ESRF, BM18, in 2022 is anticipated to provide increased resolution in volumes several times larger than human organs while using lower doses of X-rays with improved sensitivity and a much higher speed. Such fourth-generation synchrotron sources may herald new possibilities in the life sciences.

## Methods

### Sample details

Control organs were obtained from two bodies donated to the Laboratoire d’Anatomie des Alpes Françaises (LADAF). Dissections were performed respecting current French legislation for body donation. Body donation was based on free consent by the donors antemortem. All dissections respected the memory of the deceased. The post-mortem study was conducted according to the Quality Appraisal for Cadaveric Studies scale recommendations^51^. COVID-19 lung samples were obtained from the Hannover Institute of Pathology at Medizinische Hochschule, Hannover (ethics vote no. 9022_BO_K_2020). Transport and imaging protocols were approved by the Health Research Authority and Integrated Research Application System (HRA and IRAS) (200429) and the French Health Ministry.

Donor 1, from whom the heart, lung, kidney and spleen were imaged, was a 94-year-old, 45-kg, 140-cm female with right sylvian and right cerebellar stroke, cognitive disorders of vascular origin, depressive syndrome, atrial fibrillation and hypertensive heart disease, microcrystalline arthritis (gout), right lung pneumopathy (3 years before death), cataract of the left eye and squamous cell carcinoma of the skin (left temporal region).

Donor 2, from whom the brain was imaged, was a 69-year-old, 40-kg, 145-cm female with type 2 diabetes, pelvic radiation to treat cancer of the uterus, right colectomy (benign lesion on histopathology), bilateral nephrostomy for acute obstructive renal failure, cystectomy, omentectomy and peritoneal carcinoma with occlusive syndrome.

Donor 3, from whom the entire upper left lung lobe and a core biopsy from the periphery of the right upper lobe were obtained, was a 54-year-old male patient who died from COVID-19 21 d after hospitalization. Treatment involved mechanical ventilation. Regarding comorbidities, arterial hypertension and type 2 diabetes were not diagnosed before death.

Donor 4, from whom a biopsy from the lower lobe of the right lung was taken, was a male of 77 years. Resection of the lung segment was due to small pulmonary adenocarcinoma (1.4 cm), coronary heart disease, arterial hypertension and chronic rheumatic disease (polymyalgia rheumatica).

### Control organ autopsy and organ dissection (LADAF)

Bodies were embalmed with formalin solution as follows: embalming solutions (4,500 ml formalin diluted to 1.15% in a solution containing lanolin and 4,500 ml formalin diluted to 1.44%) were injected sequentially into the right carotid artery after death. Bodies were then stored at 3.6 °C. All eviscerations were performed at the LADAF between April and July 2020.

During evisceration, vessels were exposed, and surrounding fat and connective tissue were removed. Organs were post-fixed in 4% neutral buffered formaldehyde at room temperature for 1 week. The lungs were inflated with the 4% formalin solution using a large syringe to inject the solution in the bronchia until the correct lung shape was obtained.

### COVID autopsy and organ dissection

All COVID-19 autopsies were carried out according to standard procedures of the Deutsche Gesellschaft für Pathologie and followed regulatory requirements. Autopsies were performed in a room with adequate airflow (more than six air changes per hour of the total room volume) using appropriate personal protective equipment (that is, hazmat suits, boots, goggles and FFP2 or FFP3 masks). Organs were eviscerated and immediately fixed in 4% neutral buffered formaldehyde. Lungs were immediately inflated and fixed by tracheal instillation with 4% neutral buffered formaldehyde, the trachea was then clamped, and specimens were left for post-fixation in 4% neutral buffered formaldehyde at room temperature for ≥72 h before further dissection. Samples were partially dehydrated using four successive concentrations of ethanol (up to 70% ethanol, see below) before transportation to the ESRF.

COVID-19 lung samples were transported to the ESRF, Grenoble, at ambient temperature in double-sealed containers.

### Organ preservation and degassing

To ensure resistance of the samples to the X-ray dose and avoid health risks associated with formalin, all organs were equilibrated in a solution of 70% ethanol. In addition, ethanol replaces water in tissues; thus, the density of water-rich tissues and water-filled cavities decreases, while the density of other tissues remains closer to the original density. This improves X-ray contrast.

To avoid organ shrinkage, serial dehydration was performed using 50%, 60%, 75% and, finally, 70% ethanol. Each solution was 4× the volume of the organ. The time at each concentration is dependent on organ size and tissue composition (adipose tissues require longer times). In all cases, adequate dehydration can be assessed by tapping the organ container and looking for streaks in the solution. The organ that takes the longest to prepare is the brain, owing to its size and high adipose content. In our case, the brain was dehydrated over 3 weeks; however, optimization of this step has not been performed, and it is likely that this can be completed more quickly if required^8^.

Degassing was performed at each dehydration step with the organ immersed in the respective solution using a diaphragm vacuum pump (Vacuubrand, MV2, 1.9 m^3^ h^−1^) to remove both free and dissolved tissue gas.

Degassing cycles were performed with increasing duration from 2 min to 40 min (total time, typically 2 h). For each cycle, pumping was stopped when bubbling decreased in intensity or became intense enough to potentially cause organ damage. For the lung of donor 1, after each cycle, the degassed ethanol solution was gently forced into the main conducting airways with a large syringe to keep the morphology of the lung in its original inflated state.

The final degassing step was longer (typically 3–4 h) and performed until no bubbling was observed. Samples were then mounted. It should be noted that some damage attributed to degassing was observed in the brain of donor 2, and the protocol was amended to use a series of thermal cycles. The brain was immersed in a bath of pre-degassed ethanol at the appropriate concentration; this was stored at 4 °C for 5 d to equilibrate. Five cycles with successively increasing ethanol concentrations were used (50%, 60%, 70%, 70%, 70%). This protocol requires vacuum degassing only for the final mounting of the organ; however, it requires ~5 weeks. It may be possible to decrease the time by combining gentle vacuum degassing with thermal cycling.

### Organ mounting with agar–agar–ethanol gel preparation

Organ mounting requires delicacy, as any remaining gas bubbles, density inhomogeneities or insufficient sample stabilization dramatically reduce scan quality. The 70% ethanol-based mounting medium was prepared using demineralized water (20 g l^−1^ agar). Once gelled, the block was cut into small cubes of 1 cm^3^ that were immersed in ethanol at 96%, the volume ratio ensuring an equilibrium concentration of 70% ethanol (2.96 l ethanol at 96% for 1 l gel) for ~24 h. Equilibrium was assessed by the gel cubes sinking (over several dozen seconds) to the bottom of the container after agitation. Once equilibrated, the gel cubes still immersed in the solution were degassed for two cycles of approximately 1 h each until no bubbles formed.

A third of the cubes were stored in this degassed equilibrated state; the remainder were blended or more recently crushed into the ‘blended gel’, the consistency of which was adjusted by adding 70% ethanol or removing ethanol by pressing with a filter.

### Organ mounting and degassing

During the exploratory phase of this project, several mounting protocols were tested. The first method, used for the COVID-19 lung lobe, consisted of using only agarose gel cubes. This preparation allowed efficient final degassing but caused unwanted local compression of the soft organs (visible in Supplementary Video 5).

The final protocol consisted of filling the bottom few centimeters of the cylindrical container with agarose cubes to ensure the organ did not contact the container base or rotate. The container was then filled halfway with blended or crushed gel, and the organ was carefully immersed and set in the desired position (with care to avoid bubbles). The organ was covered completely with blended or crushed gel. The whole container was then degassed with care to ensure that the gel did not inflate too much due to trapped gas bubbles. After degassing for 1 h (or a few minutes for brains), the mounted organ was covered with gel cubes to ensure solid fixation. The whole mounting was degassed a final time for a few minutes, and then the container was sealed for scanning.

### Specific sample holder and mounting

We used specially designed sample holders for the scanning process. They were designed to ensure safety in case the ethanol leaked via a double sealing and to be mechanically stable and resistant to high X-ray doses. Two samples can be mounted one over the other for longer scan automation. Alternatively, only one organ can be installed, and the equivalent organ container is filled with a solution of 70% ethanol and installed in the second place for beam reference measurements in the case of local tomography scans (Fig. 1c).

### Beam properties and Hutch design

The ESRF-EBS provides a coherence length of 8 µm at 70 keV on BM05. The polychromatic beam provides a high flux while enabling a longer propagation distance before holographic imaging mode is reached. Both of these features allow longer propagation distances before reaching the geometric blurring limit due to the angular source size up to the near-field limit or a little beyond. On BM05, the propagation distance is physically limited to 3.5 m. For both scans at 6.5 and 25 µm per voxel, this restriction prevents us from reaching the theoretically estimated maximum propagation distance limit at 70 keV (6 m and 23 m, respectively), defined by the minimum between the geometric blurring of one pixel and the near-field limit; while, for 2.5 µm per voxel and lower, the propagation distance limit is 1.3 m (estimated to be close to the near-field limit).

Beam flux and energy must be tuned for each magnification and sample to ensure that the average energy is sufficient to penetrate through the sample but low enough to allow fast scans. Tuning is achieved with a combination of molybdenum and copper filters (energy tuning) and fused silica bar attenuators (flux and beam profile tuning). To preserve beam coherence, filters and optics are made of high-quality mirror polished materials to ensure material homogeneity. Similarly, X-ray optics are made of pf6/If1 beryllium, which can be highly polished. The scintillators must be as dense as possible while not degrading the resolution. LuAG:Ce provides the necessary stopping power with a high light output and the transparency required for high-resolution phase contrast. Thickness of the scintillators was optimized to provide a compromise between light output and maximum optical resolution for each X-ray optic.

All scans were performed using one of two detector optics: the d zoom (for scans at 25–6.5 µm per voxel) and the zoom optic for scans at 6–1.3 µm per voxel. Both optics were mounted with PCO edge 4.2 CLHS cameras. When designing the optics, we aimed to have the highest possible numerical aperture without having to reduce thickness of the scintillator and hence reduce resolution. The greatest limitation for the detector optics, particularly for the zoom optic, is darkening due to the polychromatic beam. To prevent darkening, the optic is either intrinsically hardened by X rays (as is the case for the d-zoom optic) or is protected from darkening through the use of a thin glassy carbon mirror to reduce internal scattering and lead glass in the front of the optic to stop as much scattering as possible. A beam stop was also used to prevent beam back scatter. Finally, we developed a rapid curing process using blue LEDs to recover optics in 12 h in case optics did darken. sCMOS detectors (2,048 × 2,048 pixels) were chosen for HiP-CT to avoid data loss during readout time and to benefit from their 74% quantum efficiency at the peak emission of LuAG:Ce. The lower full-well capacity of the sCMOS by comparison to that of CCD detectors is efficiently mitigated through our accumulation scanning protocol.

### Multi-resolution scanning protocol

One organ in its sealed container was installed in the bottom holder, and an equivalent container filled with a 70% ethanol gel was installed in the upper holder (Fig. 1c). This second container was used for acquisition of beam references. Partial angular integration of scans from the reference jar every 100 projections provided a new reference for flat-field correction of radiographs. In this manner, most of the low-frequency effects of local tomography were directly corrected, leading to normalized intensities of projections, even in case of off-center local tomography scans (Fig. 1b).

This approach also improves the dynamic range of the detector by avoiding direct exposure to the beam: the sample and its mounting medium act as filters. This protocol, termed the ‘attenuation protocol’, is derived from an approach that was originally developed to scan highly absorbing fossils, first in a monochromatic beam^24^ and later in a polychromatic beam^25^. In this protocol, the dynamic level was maximized for scanning organs at 25 µm per voxel by placing the sample slightly off axis to benefit from the natural horizontal Gaussian profile of the beam. This approach reduces the flux on the border of the sample (where the absorption is lower) and provides maximum flux to the thickest portion of the sample.

**Extended Data Fig. 6:**
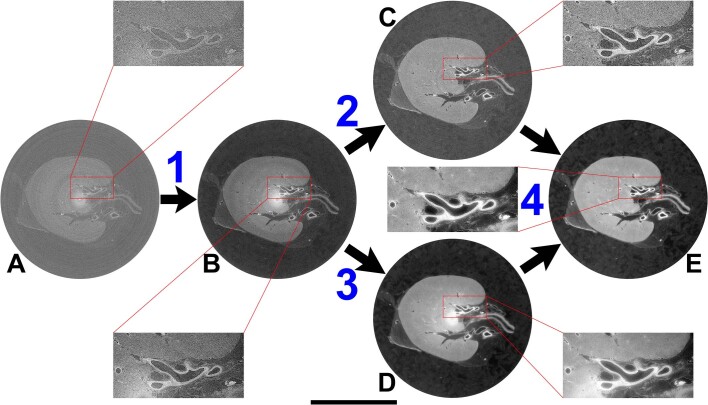
Imaging protocol developments. The attenuation scanning protocol was originally developed to image dense fossils and adapted for HiP-CT of complete human organs. In this protocol, the centre of rotation is in the middle of the cylinder used to mount the sample. There are four key principles: 1) single distance phase retrieval using Paganin et al. algorithm coupled with an unsharp mask on the radiographs after phase retrieval; 2) optimization of references absorption profile for local tomography; 3) optimization of dynamical range by adjusting detector saturation level through the sample; 4) a combination of 1, 2, 3 and the attenuation protocol adapted to organs. **A**) classical scan in edge detection mode. The saturation level of the detector is set on the beam reference without the sample **B**) Same scan reconstructed using single distance phase retrieval. **C**) Same scan but references are calculated from an equivalent jar filled with mounting media instead of using pictures of the beam without sample. **D**) Scan performed off-axis to have a lateral gradient of power in the beam to fit with absorption profile of the sample. Saturation level of the detector tuned through the sample, normal references without sample in the beam. **E**) Scan in attenuation protocol using references in equivalent jar filled with mounting media, dynamic range optimization and single distance phase retrieval. Scalebar 50mm.

As mentioned above, in addition to correction of the local tomography effect, our scanning protocol avoids direct exposure of the detector to the beam, allowing the saturation level of the detector to be adapted to the part of the sample that absorbs less instead of to the direct beam intensity. As the general absorption contrast is dominated by the cylindrical shape and the organs are close in density to that of the ethanol solution, this approach also efficiently removes any beam-hardening effect or differential phase-contrast effect in case of off-axis scans. Finally, to ensure a high dynamic level, the detector was used in accumulation^26^, with typically ten and five subframes for the scans at 25 µm per voxel and 6.5 µm per voxel, respectively. The scans at 2.5 µm per voxel and 1.3 µm per voxel were performed without accumulation to limit the X-ray dose and because their contrast is dominated by phase contrast. As phase contrast is typically 1,000 times more sensitive than absorption, it is possible to use a lower dynamic level on the detector and thus reduce the X-ray dose. The benefit of each aspect of this scanning protocol is demonstrated in Extended Data Fig.  6.

Two acquisition modes were used, depending on the size of the organ samples: half and quarter acquisitions. Most scans were performed in half-acquisition mode with the center of rotation on the right side of the field of view (moved by typically 900 pixels) to obtain a field of view of 3,800 pixels with 6,000 projections. For the largest organs, complete scans at 25 µm per voxel were performed using a quarter-acquisition protocol based on two scans (one half acquisition and one annular scan) of 9,990 projections each. Concatenation of the quarter acquisition was performed by calibrating the overlapping scan region with gradual transition and normalization of the gray levels in the area common to the two scans. Once concatenated, the reconstructed field of view was 6,000 pixels. To cover complete organs or to scan large columns in local tomography, an automated *z*-axis series was performed. The typical sampling step was 2.2 mm vertically for a corresponding beam size of 2.6 mm, corresponding to an overlap of 18% (some initial organ scans used larger steps (up to 3.6 mm) and larger overlap (up to 50%)).

All scanning parameters are presented in Table 2 and Supplementary Data 1. Based on the scan times possible (Extended Data Fig. 1), the brain supplied by donor 2 could be scanned at a full resolution of 25 µm in ~16 h (16 cm), and the organ with the smallest diameter, the kidney (7 cm), could be scanned in ~3.5 h.

### Tomographic data reconstruction protocol

Before reconstruction, radiographs obtained at 25 µm per voxel were vertically concatenated, and the remaining vertical profile of the beam (regular horizontal lines) was subtracted (at 6 µm per voxel and lower, this step is unnecessary, as the scans are not dominated by attenuation). For higher-resolution scans, we first performed tomographic reconstruction and then cross-correlation between slices to ensure alignment. For overlapping slices, we use a ponderate average to create a gradual transition from one slice to the next. This removed horizontal artifacts in these phase-dominated scans. After preprocessing, tomographic reconstruction was performed using the filtered back-projection algorithm, coupled with single-distance phase retrieval^27^ and a 2D unsharp mask on the projections as implemented in ESRF in-house software PyHST2 (ref. ^28^). All subvolumes were converted into 16 bits and vertically concatenated. The remaining ring artifacts were corrected on the reconstructed slices using an in-house MATLAB system derived from Lyckegaard et al.^29^. In some cases, final correction of horizontal stripes was performed on the reconstructed volumes after vertical reslicing.

### Histology (hematoxylin and eosin)

After HiP-CT scanning, the kidney from donor 1 was removed from the mounting jar section and stained for histological validation (*n* = 1). The coronal location of the kidney aligned with a HiP-CT high-resolution image column was manually identified, and the kidney was dissected. Large coronal sections were placed into cassettes to follow the standard tissue-treatment procedure: dehydration with a series of graded ethanol baths, paraffin embedding and microtome sectioning of paraffin blocks. Slides were routinely stained with H&E. Slides were visually inspected to identify images precisely aligned to high-resolution HiP-CT data.

**Extended Data Fig. 7:**
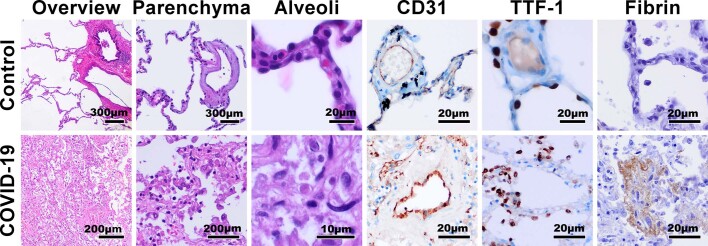
Histological staining following imaging of lung tissue of BM05. H&E and IHC staining of COVID-19 lung biopsy and control (Donor 4) lung biopsy after HiP-CT imaging. H&E staining of overview, parenchyma and alveoli in columns 1-3; CD31 (column 4), Thyroid transcription factor 1 (TTF-1 (column 5)) and Fibrin staining (column 6) (N=1). For histological comparison, representative lung samples were cut and embedded in paraffin. 2 µm thick sections were cut followed by histological staining using Hematoxylin and Eosin (HE) at the Institute of Pathology at Hannover Medical School. Immunohistochemistry for CD31 1:75 (M0823, Agilent Dako, California, USA), TTF-1 RTU (790-4756, Hoffmann-La Roche, Basel, Swiss) and Fibrin 1:500 (MABS2155, Merck Millipore, Massachusetts, USA) were stained on a VENTANA BenchMark ULTRA (Hoffmann-La Roche, Basel, Swiss) with the aforementioned dilutions and pretreatment and incubation times according to the manufacturers advice. Representative images were acquired with a Olympus CS50 camera (Olympus, Tokyo, Japan) using Olympus cellSens Software (Olympus, Tokyo, Japan) on a routine diagnostic light microscope (BX43, Olympus, Tokyo, Japan).

In addition to performing H&E staining on the kidney imaged by HiP-CT in the portions of the sample exposed to the highest X-ray dose (Fig. 3b and Extended Data Fig. 5), staining was also performed on the COVID-19 lung biopsy sample (*n* = 1) shown in Fig. 1e and on healthy control (donor 4) lung biopsies. Extended Data Fig. 7 shows H&E staining as well as anti-CD31, anti-thyroid transcription factor 1 (TTF-1) and anti-fibrin immunohistochemical staining of the lung biopsy samples. As these lung samples were biopsies, they did not receive the high radiation doses that the kidney was subjected to (see Supplementary Information for discussion and estimation of dose exposure); however, these sample underwent similar preparation and were exposed to the ESRF-EBS on BM05.

### Image analysis and statistics

Final volume renderings were performed using different 3D software packages, notably VGSTUDIO MAX 3.2.4 (Volume Graphics), Amira version 2019.6 and Dragonfly 2020.2.0. For all analyses, ImageJ version 2.1.0/1.53c Java 1.8.0_66 was used.

### Fourier shell correlation

FSC was performed on the lung sample from donor 1. Two independent scans performed sequentially (after a 0.1-mm vertical displacement of the sample and of the center of rotation) and, using the full number of projections for both, were fully reconstructed as per the normal HiP-CT procedure with the complete chain of preprocessing and postprocessing. The pairs of volumes were loaded in VGSTUDIO MAX 3.2.4 and precisely aligned. The common part was then cropped and exported in the same 16-bit TIF format, resulting into two volumes of the same size and same location but with fully independent acquisition and reconstruction. Nine randomly selected subvolumes of data at sizes ranging from 200 to 1,000 voxels were taken from both aligned volumes, and FSC was computed for each. The half-bit threshold intersection with the FSC curve was used to estimate the resolution corresponding to each voxel size. The mean and s.d. of the intercepts is provided, and FSC curves are provided in Extended Data Fig. 3.

### Comparison of image quality

For Fig. 1b and Fig. 1d, right, the SNR ratio was evaluated from the mean (*μ*) and s.d. (*σ*) of pixel values in manually drawn ROI of features (ft) and background (bg) areas. The SNR was calculated as (*μ*_ft_ − *μ*_bg_)*σ*_bg_^−1^.

In the kidney, tubule walls were used as the features, with the lumen of the same tubule used as the background region. *n* = 3 tubules per lateral resolution image were analyzed.

Comparisons of image quality were performed using the structural similarity index^30^ implemented in MATLAB 2020b (ssim function). Equally sized subvolumes from the two columns were used in their original 32-bit form (999 slices in each case). Histograms of the two subvolumes were calculated with a fixed bin width of 0.001. Skew, kurtosis and mean pixel intensity were calculated with pyradiomics version 3.0 (ref. ^52^). Structural similarity index was calculated between pairs of *xy* slices from the two subvolumes. Two images were randomly selected (by slice number); either both images were from the same subvolume (1-1 and 2-2), or the images were from different subvolumes (1-2 and 2-1). *n* = 200 pairs of images were analyzed for each group. One-way ANOVA of the output was performed in MATLAB 2020b using the anova1 function.

### Kidney glomerulus analysis

Using the scans of the whole kidney at 25 µm per voxel, the parenchyma of *n* = 1 kidney was semi-automatically segmented in Amira version 2019.6. After manually aligning all datasets at three resolutions, two cylinders, one through each of the columns at 6 µm per voxel and 1.3 µm per voxel, were defined perpendicular to the kidney surface. The cylinders had radii, lengths and volumes of 1,300 µm, 40,811 µm and 2.17 × 10^11^ µm^3^ for the dataset acquired at 6 µm per voxel and 203 µm, 9,814 µm and 1.3 × 10^9^ µm^3^ for the dataset acquired at 1.3 µm per voxel. These cylinders were considered as virtual biopsies. In the case of the dataset acquired at 6 µm per voxel, any glomeruli that fell within the cylinder were counted (blue dots in Fig. 3a, middle). For the total number of glomeruli, the volume of parenchyma in the virtual biopsy at 6 µm per voxel was calculated by semi-manual segmentation in Dragonfly 2020.2.0. The number of glomeruli counted in the virtual biopsy at 6 µm per voxel was divided by the volume of parenchyma in the virtual biopsy at 6 µm per voxel and multiplied by the total volume of the parenchyma. In the dataset acquired at 1.3 µm per voxel, any glomeruli that touched the cylinder were manually segmented in Amira version 2019.6 in 3D. Volume, surface area and sphericity were calculated with the binary mask from these segmentations in ImageJ using the MorphoLibJ plugin (for analysis of 3D regions)^53,54^. Alignment of HiP-CT and H&E histology sections was performed in VGSTUDIO MAX 3.2.4. Pseudocoloring of HiP-CT data was performed by applying a filter of a median of 100 pixels on the histological slice and superimposing these colors onto the original gray-level slice of the HiP-CT data. The gray-level version of the histological slice was obtained by desaturating the picture, inverting its contrast and linearly adjusting the contrast to reach 0.2% of saturation in black and white on an 8-bit scale.

### Lung microstructure analysis

Manual segmentation of secondary pulmonary lobules was performed in Amira 2019.6 using scans of the COVID-19 upper right lung lobe performed at 25 µm per voxel. Data were binned to 50 µm per voxel before segmentation to reduce computational load. Acinus segmentation was performed using a column of data acquired at 6 µm per voxel in the control lung and a region of the COVID-19 lung selected by a pulmonary radiologist as relatively spared. Regions of the image that contained a clear terminal bronchiole were cropped, and segmentation was performed semi-manually: first, tissue–air boundaries were enhanced using an unsharp mask and a Canny edge filter in ImageJ^53^, enhanced images were then transferred to Amira 2019.6, and a 3D magic wand tool was used to segment the 3D airspace. For microstructural analysis, *n* = 6 250 × 250 × 250-pixel subvolumes from the datasets of the COVID-19 and control lung acquired at 2.2 µm per voxel were chosen (randomly in the case of the control lung and, for the COVID-19 lung, randomly within larger areas designated by pulmonary histopathologists as structurally preserved or consolidated, that is, from areas where alveolar morphology was identifiable versus areas where there was widespread consolidation of the parenchyma). Binary images separating airspace from alveolar septa and microvessels were produced in ImageJ by thresholding the images using one of the two following methods: the ‘triangle’ method for control and COVID_S_ data and the ‘Yen’ method for COVID_C_ data^53^. A 3D Chamfer distance calculated from the MorphoLibJ plugin^54^ was used (using Svensson 3, 4, 5, 7) to create the distance maps (Fig. 4d(i–iii)). The BoneJ plugin (version 2) was used to perform morphological analysis including connectivity, surface area-to-volume ratio, airway diameter and septal thickness^55,56^. One-way ANOVA with Tukey’s comparison was used for statistical analysis of microstructural measures.

### Reporting Summary

Further information on research design is available in the Nature Research Reporting Summary linked to this article.

## Online content

Any methods, additional references, Nature Research reporting summaries, source data, extended data, supplementary information, acknowledgements, peer review information; details of author contributions and competing interests; and statements of data and code availability are available at 10.1038/s41592-021-01317-x.

## Supplementary information

Supplementary Information Supplementary Fig. 1 and Tables 1 and 2

Reporting Summary

Supplementary Video 1 Multiscale HiP-CT imaging of an intact human brain.

Supplementary Video 2 Multiscale HiP-CT imaging of an intact human lung.

Supplementary Video 3 Multiscale HiP-CT imaging of an intact human kidney.

Supplementary Video 4 Anatomically labeled 2D slices and comparative histology from multiscale HiP-CT imaging in five human parenchymal organs.

Supplementary Video 5 Multiscale HiP-CT imaging of an intact human lung lobe from a patient who died from COVID-19-related ARDS.

Supplementary Data 1 Scan parameters for all samples.

## Data Availability

Image data that support the findings of this study are publicly available from the ESRF data repository (https://human-organ-atlas.esrf.eu). Individual DOIs for all datasets are listed in Supplementary Table 2. Source data are provided with this paper.

## Source data

Source Data Fig. 1 Data for box plots and lateral resolution correlation.
Source Data Fig. 3 Glomerular statistics.
Source Data Fig. 4 Data for box plots of lung microstructure in Fig. 4d(vi,vii).
Source Data Fig. 4 Data for the histogram of lung airway diameter in Fig. 4d(ix).

## Code availability

HiP-CT data were reconstructed using custom code written in MATLAB 2017 available at https://github.com/HiPCTProject/Tomo_Recon and the PyHST2 software package (https://software.pan-data.eu/software/74/pyhst2). Volume renderings were performed using 3D software packages, notably VGSTUDIO MAX 3.4 (Volume Graphics), Amira version 2019.6 and Dragonfly 2020.2.0. Comparisons of image quality were performed using MATLAB 2020b (ssim function). ImageJ version 2.1.0/1.53c Java 1.8.0_66 and specific plugins (BoneJ version 7.0.11 and MorphoLibJ version 1.4.2.1) were used for lung morphology analysis; pyradiomics version 3.0 (https://pyradiomics.readthedocs.io/en/latest/index.html) was used to calculate skew and kurtosis of image histograms. FSC analysis code was slightly modified from https://github.com/bionanoimaging/cellSTORM-MATLAB/blob/master/Functions/FSC.m.
