# Imaging intact human organs locally resolving cellular structures using hierarchical phase-contrast tomography

**DOI:** 10.1038/s41592-021-01317-x

**Published:** 2021-11-04

**Authors:** C. Walsh, P. Tafforeau, W.L. Wagner, D.J. Jafree, A. Bellier, C. Werlein, M.P. Kühnel, E. Boller, S. Walker-Samuel, J.L. Robertus, D.A. Long, J. Jacob, S. Marussi, E. Brown, N. Holroyd, D.D. Jonigk, M. Ackermann, P.D. Lee

**Affiliations:** 1 Department of Mechanical Engineering, University College London, U.K; 2 Centre for Advanced Biomedical Imaging, University College London, U.K; 3 European Synchrotron Radiation Facility, Grenoble, France; 4 Department of Diagnostic and Interventional Radiology, University Hospital Heidelberg, Heidelberg, Germany; 5 Member of the German Lung Research Centre (DZL), Translational Lung Research Centre Heidelberg (TLRC), Heidelberg, Germany; 6 Developmental Biology and Cancer Programme, Great Ormond Street Institute of Child Health, University College London, UK; 7 UCL MB/PhD Programme, Faculty of Medical Sciences, University College London, UK; 8 French Alps Laboratory of Anatomy (LADAF), Grenoble Alpes University, Grenoble, France; 9 Institute of Pathology, Hannover Medical School, Hannover, Germany (Carl-Neuberg-Straβe 1, 30625 Hannover); 10 Member of the German Center for Lung Research (DZL), Biomedical Research in Endstage and Obstructive Lung Disease Hannover (BREATH); 11 Department of Histopathology, Royal Brompton and Harefield NHS Foundation Trust, London, UK; 12 National Heart & Lung Institute, Imperial College London, London, UK; 13 Centre for Medical Image Computing, University College London, London, UK; 14 UCL Respiratory, University College London, London, UK; 15 Institute of Functional and Clinical Anatomy, University Medical Center of the Johannes Gutenberg University Mainz, Mainz, Germany; 16 Institute of Pathology and Department of Molecular Pathology, Helios University Clinic Wuppertal, University of Witten–Herdecke, Wuppertal, Germany

## Abstract

Imaging intact human organs from the organ to the cellular scale in three-dimensions is a goal of biomedical imaging. To meet this challenge, we developed Hierarchical Phase-Contrast Tomography (HiP-CT), an X-ray phase propagation technique utilising the European Synchrotron Radiation Facility’s Extremely Brilliant Source (ESRF-EBS). The spatial coherence of the ESRF-EBS combined with our beamline equipment, sample preparation and scanning developments, enabled us to perform non-destructive, 3D scans with hierarchically increasing resolution at any location in whole human organs. We applied HiP-CT to image five intact human organs: brain, lung, heart, kidney and spleen. HiP-CT provided a structural overview of each whole organ followed by multiple higher resolution volumes of interest, capturing organotypic functional units and certain individual specialised cells within intact human organs. We demonstrate the potential applications of HiP-CT through quantification and morphometry of glomeruli in an intact human kidney, and identification of regional changes to the tissue architecture in the lung of a deceased COVID-19 donor.

Biological tissues are complex three-dimensional (3D) structures arranged hierarchically from individual specialised cells to organotypic functional units, e.g. alveoli in the lung, up to intact, whole organs. Spatial relationships, 3D morphology and interaction within and across these length scales collectively provide a basis for biological function. Thus, mapping of the spatial organisation and morphology of individual cells up to the scale of intact organs is fundamental to understanding system-level behaviours in health or disease.

Spatial mapping at the single cell level for entire human organs is presently unfeasible in terms of existing techniques, data storage requirements and analysis or interpretability^1^. A more practical approach is to provide overall spatial context at a lower resolution, then use this data to select smaller regions for higher resolution imaging; this type of imaging can be considered hierarchical imaging. Currently hierarchical imaging typically involves physical sub-sampling of larger samples, before high-resolution imaging^2^. Physical sub-sampling brings challenges in data registration and the requirement that correct, or representative, subsamples are collected^2,3^. Novel 3D imaging techniques are therefore required to bridge length scales from cellular level spatial relationships to the architectural organisation of intact human organs.

In recent years there has been progress towards achieving 3D imaging of intact organs at multiple length scales. One approach is optical clearing, which homogenises refractive index in biological tissues, allowing high-resolution 3D imaging modalities such as light-sheet microscopy or optical projection tomography. These have allowed the visualisation of cellular structures in human embryonic development^4,5^ and mouse models of cancer metastasis^6^ and drug uptake^7^. Optical clearing of whole adult human organs has recently been achieved; however, it requires several months for intact organs, causes changes in tissue morphology, and light-sheet microscopes cannot presently image whole organs in their intact state^8^. High resolution magnetic resonance imaging (MRI) requires minimal tissue preparation, does not cause tissue distortion and achieves 100 μm voxels in an *ex vivo* human brain^9^. However, this type of MRI scan requires 100 hours for the human brain and does not achieve the resolution required to capture cellular detail. Multi-beam electron microscopy can provide images of human tissue from cellular to sub-cellular scales^2^, but cannot capture the large tissue volumes required for whole human organs.

Synchrotron X-ray tomography (sCT) is a promising approach to image whole human organs at cellular detail^10,11^. X-rays are intrinsically suited to imaging different length scales due to their high penetration and short wavelength. Some specialised synchrotron tomography instruments have achieved high resolution relative to the overall size of the biological sample: including resolving cellular features in ⌀ 32 mm human spinal cord biopsies^12^, achieving ~5 μm resolution in human lung biopsies (⌀6 mm)^13^, and 87 nm resolution in volumes of interest (VOIs) within a ~⌀1 mm intact drosophila leg^14^. In these cases, the entire sample was scanned at the reported resolutions rather than a hierarchical series of resolutions performed with a single instrument. Local tomography or zoom tomography is an established synchrotron technique that enables this hierarchical approach. Whilst it has been developed for use in small (500 μm) biological tissue samples, contrast agents such as barium or osmium are often used^1,15,16^. For large objects, where staining is far more challenging, local tomography has been confined to objects with high density differences, such as those found in bones or fossilised remains^17–19^. There are no established sCT techniques that achieve cellular resolution far from the central axis of rotation in large soft tissues such as human organs. Phase-contrast based sCT^18,20,21^, specifically, propagation-based imaging^22^, can theoretically be applied to image large, soft tissues. However, this requires a high energy X-ray beam to penetrate large samples, coupled with the high coherence and flux required to resolve cellular detail (and a large enough beam size to scan whole organs in reasonable timeframes), which was previously not available in any laboratory source or at a single synchrotron beamline worldwide.

Recently, the first high-energy (6 GeV), 4^th^ generation, synchrotron source at the European Synchrotron Radiation Facility (ESRF), named the Extremely Brilliant Source (EBS), has provided the beam coherence required to resolve faint density contrasts at high-resolution whilst achieving a one-hundred-fold increase in brilliance compared to its predecessors^23^. We have leveraged the latest iteration of the ESRF-EBS, using the test beamline BM05, to develop a technique termed Hierarchical Phase-Contrast Tomography (HiP-CT). We demonstrate the utility of HiP-CT to image intact human tissues across length-scales, spanning whole organs down to some specialised cells in VOIs. We apply HiP-CT to image a series of intact human organs and provide quantitative and morphometric insights into healthy human kidney and lung. We additionally apply HiP-CT to a contemporary biomedical problem by characterising changes to the architecture of lungs from a patient with fatal COVID-19.

## Results

### HiP-CT development and implementation

**Figure 1:**
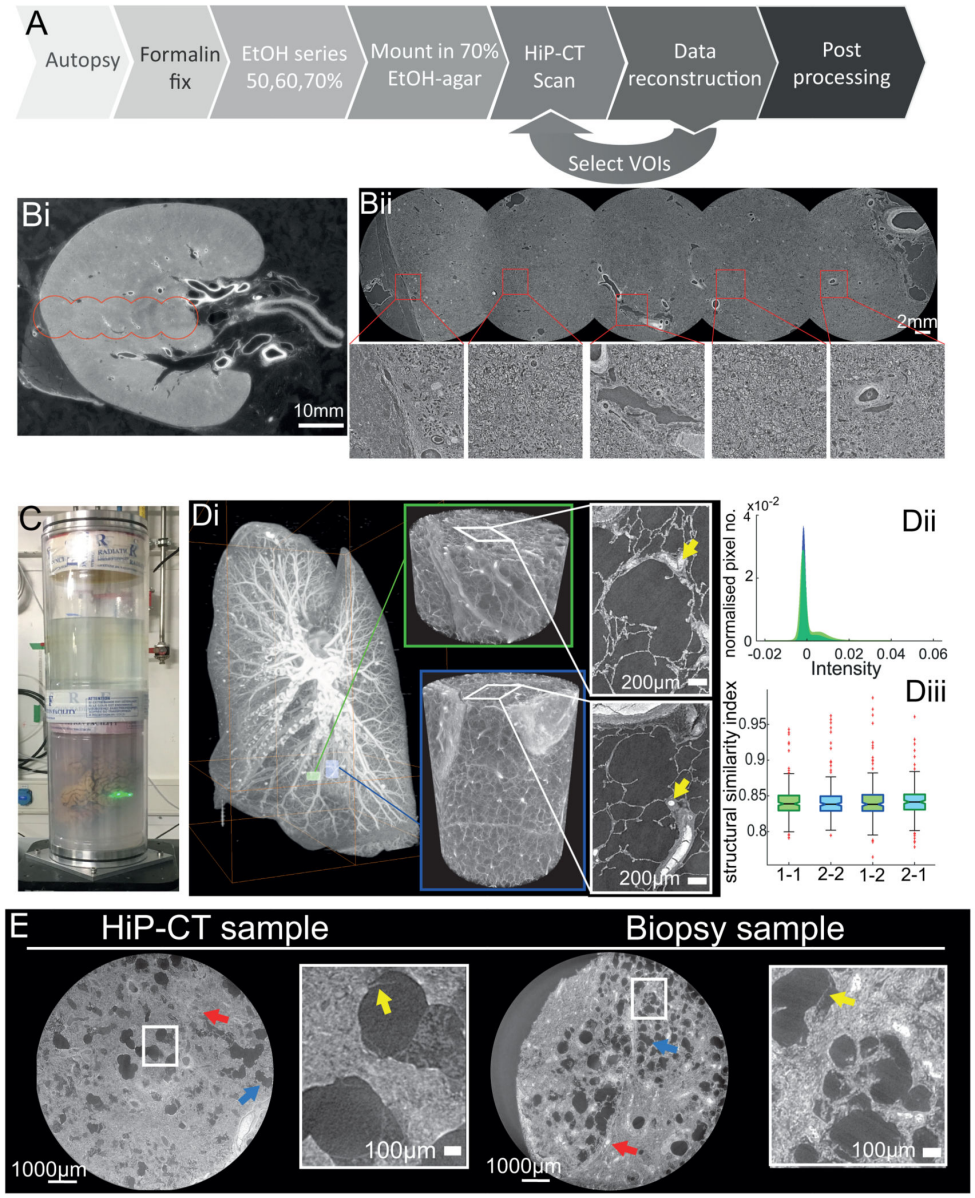
A HiP-CT pipeline for multiscale 3D imaging from whole organ to cellular resolution within large intact soft tissue samples. **A**) Flow chart of HiP-CT sample preparation and imaging procedure, the ability to select specific higher resolution scan regions based on lower resolution scans provides hierarchical tissue structure images in a data efficient manner. Bi) 2D image slice (25 μm/voxels) showing the location of a series of 2.5 μm/voxel which transect the organs radius (red circles). **Bii**) HiP-CT scans at 2.5 μm/voxel every 7 mm from the external kidney surface (left) to the centre of the sample (right). The scans are overlapped and stitched to provide a complete organ. The zoom-in show constant level of data quality and precision over the complete transect through the use of the reference scan procedure. **C**) Photograph of the intact human brain mounted in the PET jar with the ethanol-agar stabilization and reference jar on top. **Di**) Maximum intensity projection of whole human lung with two randomly selected VOIs imaged at 2.45 μm voxel resolution shown in green (VOI1) and blue (VOI2). 3D reconstructions of the two high-resolution VOIs are shown, with 2D slices in the insets. In the 3D high resolution VOIs the fine mesh of pulmonary blood vessels, complex network of pulmonary alveoli and their septae can be seen. Yellow arrows denote occluded capillaries in 2D slices **Dii**) Image stack histograms for the green (VOI1) and blue (VOI2) high-resolution VOIs respectively (fixed bin width = 0.0001). Intensity distributions are comparable with positive skew (1.82 and 2.68) and kurtosis (6.44 and 11.88) for VOI1 and VOI2 respectively, histogram intersection is 71% ±3% for fixed bin widths range 1×10^-2^-3×10^-4^. **Diii**) Box whisker plot showing the structural similarity index between n = 200 pairs of 2D slices independently sampled either from within the same VOI (1-1 and 2-2) or from different VOIs (1-2 and 2-1) for each group respectively; one-way ANOVA (two-sided); *p* = 0.8765, df = 3, F = 0.23). Boxplots show median = box centreline, interquartile range (75-25 percentile) of data = box bounds, data range excluding outliers = whiskers, values more than 1.5 times the interquartile range above or below box bounds are denoted as outliers – red crosses. E) Single representative slices of high-resolution scans from a HiP-CT image of an intact whole human lung lobe affected by COVID-19 (Donor 3) and a biopsy taken from the same patient’s contralateral lung. Both VOIs are captured from the upper peripheral region of each upper lung lobe. In HiP-CT images, fine structure of the tissue including the blood capillaries (red arrows) and alveoli (blue arrows) as well as thin alveolar septae (yellow arrows in insets) are depicted.

HiP-CT requires specific sample preparation, scanning and reconstruction approaches shown in Figure 1A (see Online Methods for detailed steps). Briefly, organs are fixed, partially dehydrated and physically stabilised for scanning (Figure 1C). HiP-CT scans are then performed hierarchically, the first typically at 25 μm/voxel over the whole organ followed by VOIs at 6.5 μm/voxel and 1.3-2.5 μm/voxel. Scanning time for HiP-CT is faster than other techniques^8,9^, currently ~16 hrs for a whole brain at 25 μm/voxel and ~3.5 hrs for a whole kidney at 25 μm/voxel (see Online Methods and Extended Data 1) for further details). Our experimental setup is largely automated and can be adjusted to increase scanning speed for smaller organs whilst accommodating different fields of view for higher resolution imaging. Our scanning procedure allows users to select high-resolution VOIs with the spatial context provided by the preceding lower resolution scans (see Supplementary Information).

Long term physical stabilisation of the organs was necessary for HiP-CT, both to enable multi-resolution registration and to allow organs to be removed from the beamline whilst higher resolution VOIs were selected. Our optimised organ stabilisation method (see Online Methods for further details) using agar-agar gels was highly effective for maintaining organs positions (see Supplementary Video 1 where the higher resolution scans were taken several months after the lower resolution scans and simply registered by manual rigid transformation) and uses easily obtained materials. Several other key procedures and modifications of the BM05 experimental hutch, summarised and prioritised in Table 1 and outlined in Online Methods, describe the essential developments for HiP-CT. Central to HiP-CT is the increased spatial coherence provided by the EBS. This spatial coherence allows propagation distances approaching the near field limit for the highest resolution scans and thus resolves the faint density contrasts in soft tissue^1^.

Much of the HiP-CT scanning geometry aims to reduce X-ray dose to the sample, optimise the detector dynamic range, reduce ring or bubble artefacts and supress beam hardening. Matching the horizontal gaussian profile of the beam to the attenuation profile of the sample at 25 μm/voxel, achieves all the above benefits (see Online Methods for details). The dose limit is crucial in HiP-CT, as once it is surpassed bubbling is induced in the sample causing tissue damage and introducing artefacts (see Supplementary Information for further details).

To perform the extreme off axis local tomography reconstructions, we have developed the use of hierarchical reference scans. For every higher resolution VOI a reference scan is taken in an identical agar-ethanol filled jar mounted on top of the sample jar (see Figure 1C). This scan provides a background estimate that can be removed when performing reconstruction, thus eliminating much of the low frequency sinusoidal background variations. To demonstrate the effectiveness of the reference scan method, we performed a series of high-resolution scans transecting the kidney from the centre of rotation to the outer edge (Figure 1Bi). The consistent image quality across the high-resolution scans (Figure 1Bii) can be appreciated in the zoomed insets and no correlation between signal-to-noise ratio (SNR) and lateral scan position (Pearson’s correlation coefficient -0.25 p = 0.21, see Extended Data 2).

The HiP-CT scanning procedure adapts two protocols originally developed to image large fossils. The first, the attenuation protocol, normalizes the absorption in the field of view^24,25^ whereas the second, the accumulation protocol, provides extended dynamic range^26^. At 25 mm/voxel, HiP-CT scans are dominated by attenuation, hence there is reduced contrast sensitivity and a requirement for more accumulations, whereas higher resolutions are phase dominated leading to fewer accumulations (see Table 2, and Supplementary Data 1 for all scanning parameters). The dominance of attenuation at lower resolution creates horizontal line artefacts during concatenation of the radiographs, due to the vertical profile of the beam. To remove these artefacts the vertical profile residual background after flatfield correction is subtracted before reconstruction. For higher resolution scans where phase effects dominate, this step is not required (see Online Methods for details).

After pre-processing of radiographs to generate high-quality local tomography (see Online Methods for further details), image reconstruction can be performed using a filtered back-projection algorithm, coupled with a single-distance-phase-retrieval^27^, combined with a 2D unsharp mask performed on the projection phase maps, as implemented in the software PyHST2^28^. All the sub-scans (covering typically 2.5 mm vertically) are concatenated after reconstruction. Subsequently, residual ring artefacts that would not have been removed in the preceding steps are corrected on reconstructed slices^29^.

To assess the performance of HiP-CT for imaging human organs, we scanned the intact human lung of a 94-year-old female (Donor 1 – clinical details in Online Methods) beginning with the whole organ at 25 μm/voxel, before selecting VOIs for zoomed imaging at 6.5 μm/voxels and 2.5 μm/voxels (green and blue columns of Figure 1Di).

We estimated the resolution of HiP-CT via Fourier shell correlation analysis (FSC)^1,16^. Resolution was estimated at the 1/2 bit criterion to be 10.4±0.17 μm for 2.5 μm/voxel images, 18.3±0.6 μm for 6.5 μm/voxel and 72±3.4 μm for 25 μm/voxel images (see Extended Data 3). To assess the consistency in the quality of higher resolution scans at different tissue depths and distances from the rotational centre of the intact organ, we analysed intensity distributions across two high resolution VOIs. We found the image intensity histograms had an intersection of 71±3 %. Both distributions showed positive skewing and kurtosis with minimal differences in mean intensities between the different VOIs and (Figure 1Dii). In addition, we quantified image quality differences between the two volumes using the structural similarity index (SSI)^30^ (Figure 1Diii). No significant difference in SSI (median values; 0.839-0.841, *p* = 0.88) was observed across compared image volumes, indicating that HiP-CT can achieve high resolution scanning in any region of the intact human lung with consistent quality.

The large size of adult human organs is a fundamental imaging challenge: the penetration depth of the imaging photons (X-ray for HiP-CT) and diffusion of reagents (ethanol for HiP-CT) may both be limiting factors. Accordingly, image quality tends to decrease from small to large specimens. We therefore qualitatively compared high zoom HiP-CT scans of intact human lung and a physically subsampled biopsy (cylindrical biopsy ⌀8.1 mm height 14 mm). The image quality of the two samples was found to be indistinguishable (Figure 1E). The fine structure of the lung tissue including the capillaries and alveoli are depicted in a histopathologically correct and non-distorted manner with very thin membranes within the alveolae (yellow arrows) being some of the smallest structures (~5 μm thickness) evident in both samples.

Thus HiP-CT is versatile, providing high quality imaging of human lungs across multiple length scales, independent of the region sampled or the size of the tissue imaged.

### HiP-CT imaging of whole human organs

**Figure 2:**
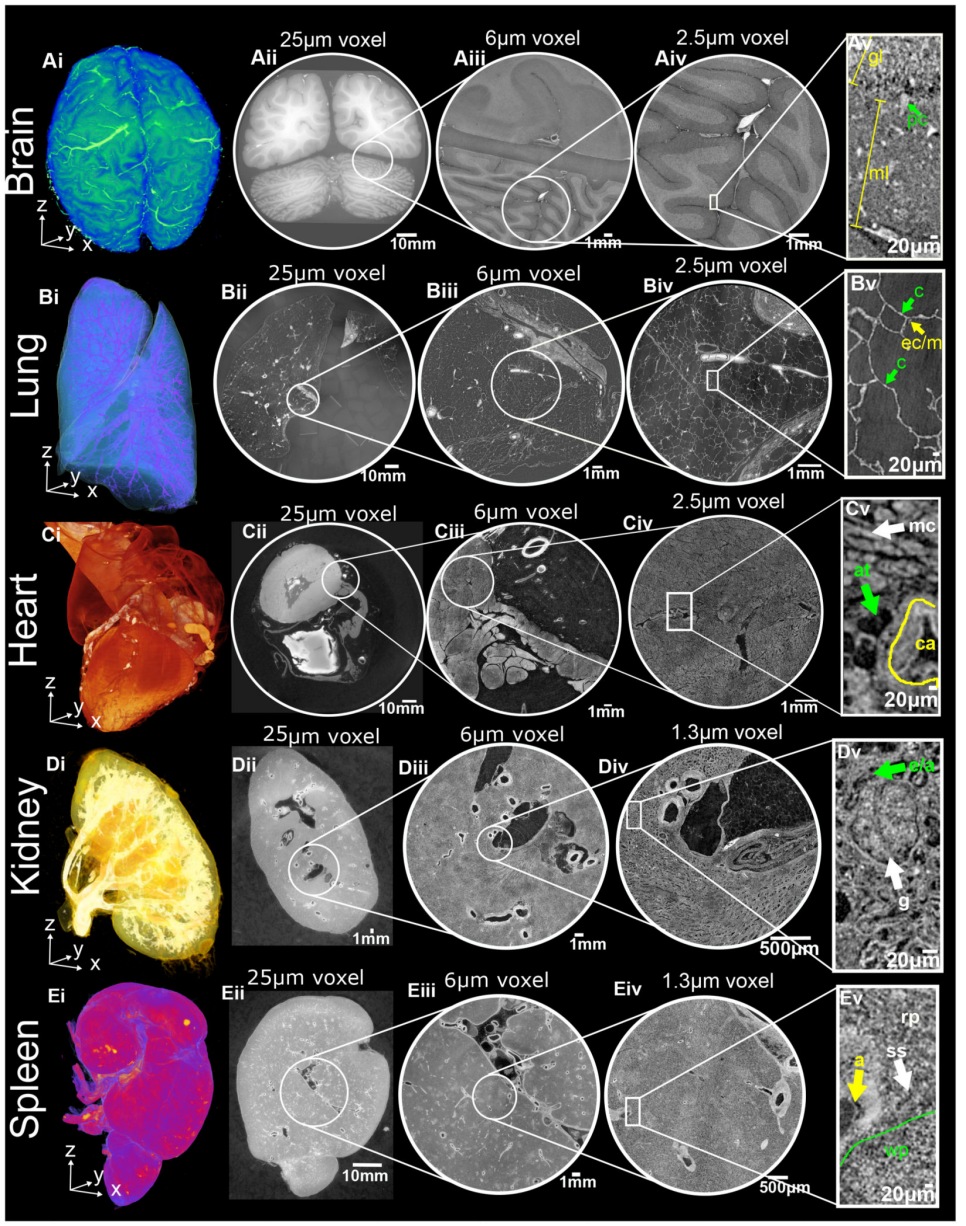
HiP-CT enables 3D imaging of organotypic functional units across intact human organs. HiP-CT of brain (**A**), lung (**B**), heart (**C**) kidney (**D**) spleen (**E**), where for each organ (**i**) shows 3D rendering of the whole organ using the 25 μm/voxel scans. Subsequent 2D slices (**ii-iv**) show the posisitons of the higher-resolution VOIs relative to the previous scan. (**v**) Shows a digital zoom of the highest resolution image with annotations depicting characteristic structural features: in the brain (molecular layer, ml; granule cell layer, gl; Purkinje cell, pc), in the lung (blood capillary, c; epithelial cell/macrophage, ec/m), in the heart (myocardium, mc; coronary artery, ca; adipose tissue, at), in the kidney (efferent/afferent arteriole, e/a; glomerulus, g) and in the spleen (red pulp, rp; white pulp, wp; arteriole, a; splenic sinus, ss). All images are shown using 2x binning.

Next, we sought to apply HiP-CT to image multiple length scales in a variety of human organs. Intact lung, heart, kidney and spleen (Donor 1) and brain (Donor 2), were acquired from two donors (clinical details in Online Methods), processed and imaged by HiP-CT. Images were sequentially acquired at 25 μm/voxel to capture the entirety of each organ, before 6 μm/voxel and 2.5 or 1.3 μm/voxel scans of selected VOIs, (Supplementary Videos 1–4). At 25 μm/voxel, macroscopic features of each organ are unambiguously identifiable through anatomical location and morphology including: sulci and gyri of the cerebral cortex (Figure 2Ai and Aii, Supplementary Video 1 and 4), individual lobules of the lung (Figure 2Bi and Bii, Supplementary Video 2 and 4), the four chambers of the heart and associated coronary arteries (Figure 2Ci and Cii and Supplementary Video 4), the pelvis and calyces of the kidney (Figure 2Di and Dii, Supplementary Video 3 and 4) and the pulpa of the spleen (Figure 2Ei and Eii and Supplementary Video 4).

Human organ function is driven by the collective activity of organotypic functional units. Each functional unit comprises multiple spatially arranged cell-types that facilitate specialised functions. We used high-resolution HiP-CT to image functional units across the same five human organs (Figure 2). The hierarchical nature of HiP-CT, isotropy of the image data and high resolution facilitate the identification of specific organotypic structures and in certain cases specialised cells. The use of comparative and correlative histology is a further aid for distinguishing specific structures and cell types (see Supplementary Video 4). In the brain, the layers of the cerebellum are visible (Figure 2Aiii, 2Aiv) and a number of individual Purkinje cells are evident between the molecular and granule cell layers, identifiable by their distinctive pyramidal cell body and anatomical location^31^ (Figure 2Aiv and Av and Supplementary Video 4). SNR of 4 Purkinje cells was 6 ± 1.6 (Extended Data 4). In the lung the intralobular septa and septal veins are visible in (Figure 2Biii) along with the terminal bronchi which lead into acini (Supplementary Video 2 and 4). Within the acini cup-shaped alveoli across which gas exchange occurs, brightly contrasted cell-sized objects mainly at intersections of the alveolar septae are evident, that we can identify as type II pneumocytes and / or alveolar macrophages based on comparative histology (Figure 2Biv, Bv and Supplementary Video 4). Bundles of cardiac muscle fibres are visible in the heart (Figure 2Ciii), consisting of individual cardiomyocytes, which can be distinguished through their distinctive shape, arrangement in fascicles and via comparative histology^32^ (Figure 2Civ and Cv, Supplementary Video 4). In the kidney, epithelial tubules comprising the nephron are evident (Figure 2Diii) which, at their apex, harbour the intricate capillary network of the glomerulus (Figure 2Div, Dv and Supplementary Video 4), specialised for the filtration of blood. Finally, the organization of red and white pulp in the spleen (Figure 2Eiii) are visible, the former containing splenic sinuses and the latter containing periarterial lymphoid sheaths (PALS) and lymphocyte-rich follicles (Figure 2Eiv, Ev and Supplementary Video 4). Collectively, these images show that HiP-CT is capable of imaging intact human organs down to the resolution of organotypic functional units and certain types of specialised cells.

### Quantitative HiP-CT in healthy human kidney

Next, we sought to assess the utility of HiP-CT to obtain physiologically relevant structural information using the human kidney as an example. The functional capacity of the human kidney arises from the collective activity of individual units called nephrons. Each nephron has a glomerulus: a network of blood capillaries which is the site of blood filtration. The total number of glomeruli (N_glom_) is indicative of the kidney’s capacity for filtration, and as nephrons are not (re-) generated through the life of an individual^33^, reduction in N_glom_ is a feature of physiological ageing and chronic kidney disease^34–36^.

**Figure 3:**
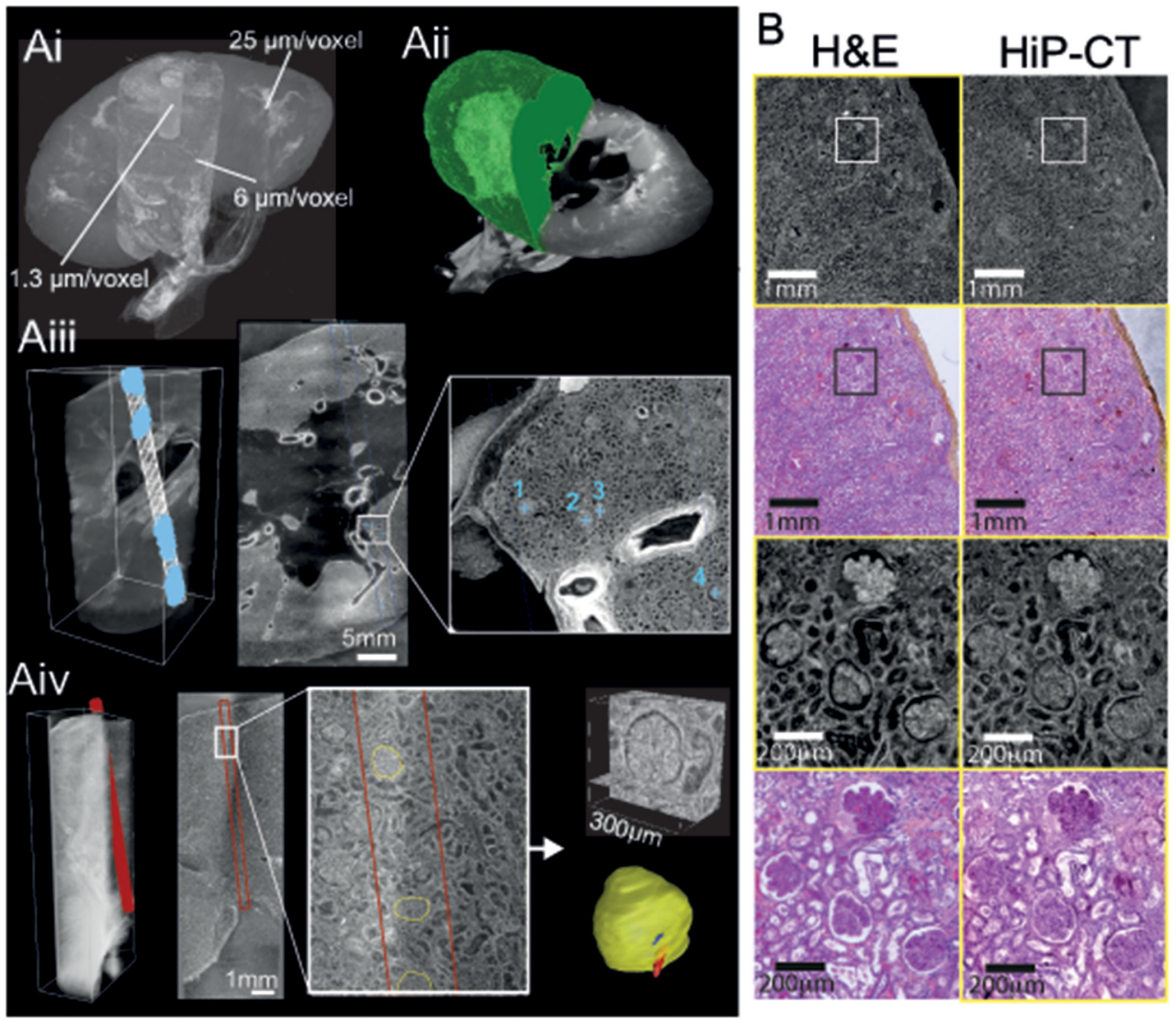
HiP-CT analysis of kidney to measure glomeruli morphology and nephron number. Ai) The three resolution (25, 6 and 1.3 μm/voxel) datasets of HiP-CT taken of a human kidney aligned and overlaid. Aii) shows the measurement of the parenchymal volume semi-automatically segmented (green). Aiii) shows the 6 μm/voxel dataset with the virtual biopsy cylinder in white, the parenchymal volume within the cylinder was measured. A representative 2D slice with inset shows 4 labelled glomeruli. In total 853 glomeruli that were within the cylinder were counted, a blue cross was used to denote the approx. centre of each glomerulus. Aiv) the 1.3 μm dataset with virtual biopsy (red cylinder) shown. The 13 glomeruli within this cylinder were segmented in 3D as shown in the 2D representative slice with inset. B) Comparison of HiP-CT with an aligned histopathological sections (n = 1) (Haematoxylin and Eosin (H&E) stained) taken after all HiP-CT scanning was finished. The left-hand column shows light micrographs of H&E stained histopathological sections and the right-hand column shows 2D tomograms of HiP-CT, yellow boxes denote images that have been pseudo-coloured (from HiP-CT) or converted in grey levels and inverted in contrast (from histological observations).

We assessed N_glom_ in a 94-year-old female (clinical information in Online Methods – **Donor 1**) with HiP-CT (**Supplementary Video 3** and Figure 3Ai). At 25 μm/voxel, the parenchymal volume was segmented (Figure 3Aii), at 6 μm/voxel, the number of glomeruli within a representative volume were counted (Figure 3Aiii). N_glom_ was calculated as 310,000 for this individual, which is within the range of previous studies using either stereological analysis^34,36^, contrast-enhanced MRI^37^ or CT^35^ and specifically accords well with measures of N_glom_ in older individuals^36,38^.

Using 1.3 μm/voxel scans we also assessed glomerular volume, an important structural determinant of overall kidney health which has been shown to increase in individuals with reduced nephron number, obesity and hypertension^34,36^. Thirteen glomeruli were manually segmented to calculate V_glom_ (Figure 3Aiv) resulting in a value of 5.05 ± 0.09 × 10^-3^ mm^3^, similar to the range of V_glom_ (3-5 × 10^-3^ mm^3^) estimated by stereological and MRI analysis^36,37^. Additionally, the isotropy of HiP-CT enabled the calculation of mean glomerular surface area (1.7 ± 0.4 × 10^5^ μm^2^) and sphericity (0.58 ± 0.09), both of which cannot be assessed using conventional modalities^38^. Moreover, we matched microscopy of histological sections to the same regions of the kidney imaged with HiP-CT, finding an equivalence of structural detail in the two modalities (Figure 3B). Histological results validate the accuracy of glomeruli segmentation and show that sample preparation and high X-ray dose expose during HiP-CT do not cause evident morphological tissue damage (see Extended Data 5). Therefore, HiP-CT has the potential to quantify functional units and their 3D morphology with histological resolution within intact human kidneys, providing morphometric insights across entire organs.

### Quantitative HiP-CT characterisation of SARS-CoV-2 lung

We finally sought to apply HiP-CT to a contemporary biomedical problem by examining the structural changes in the lungs of a patient diagnosed with COVID-19, caused by severe acute respiratory syndrome coronavirus 2 (SARS-CoV-2). The major cause of morbidity and mortality in COVID-19 is severe acute respiratory distress syndrome (ARDS) ^39^. The clinical course of COVID-19 is well described ^40^ and established histological features of SARS-CoV-2-infected lungs include alveolar inflammation, fibrosis and necrosis ^41^. Recently, these findings were built upon by sCT and 3D reconstruction of millimetre-thick samples from COVID-19 lungs, demonstrating hyaline fibrotic deposits, lymphocytic infiltrates and vasculature occluded by thrombi ^42^.

**Figure 4:**
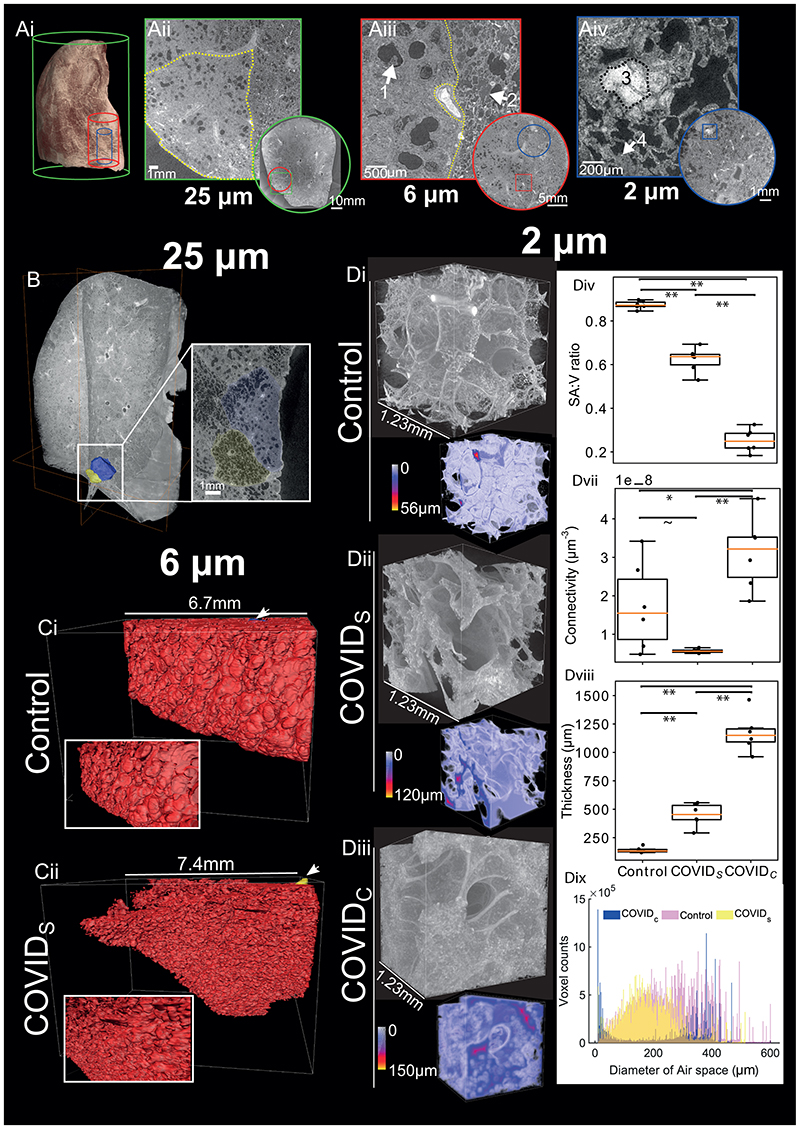
HiP-CT with 3D image analysis and morphometry in the lung of a patient with COVID-19 **Ai**) 3D reconstruction from 25 μm/voxel HiP-CT scanning of the intact upper left lung lobe from the autopsy of a patient deceased from COVID-19-related ARDS. The high-resolution VOIs are shown in red (6.5 μm/voxel) and blue (2.5 μm/voxel). **Aii**) At 25 μm/voxel, high intensity regions are observed in the lung periphery. The yellow dashed line delineates a secondary pulmonary lobule. **Aiii**) At 6.5 μm/voxel, heterogeneity in the lung parenchyma including: (1) dilated alveolar ducts and diffuse loss of alveolar structural organisation; and (2) comparatively well-preserved alveolar structure with some oedematous changes. **Aiv**) At 2.5 μm/voxel we observed (3) alveolar obstruction, likely representing clotted blood based on its high intensity; and (4) interstitial thickening of alveolar septae. **B**) 3D reconstruction of COVID-19 lung with segmentation of two adjacent secondary pulmonary lobules with differing degrees of parenchymal deterioration. **Ci**) 3D reconstruction of segmented acini structure within the SARS-CoV-2-uninfected (control) lung and **Cii**) the SARS-CoV-2 infected lung. **Di**)-**Diii**). 3D reconstruction of representative VOI at the high resolution (2.5 μm voxels) for the control, COVIDs and COVID_C_ groups respectively. Duplicated volumes show visual representations of the airspace-tissue interface in COVID-19 lung, where a smaller distance between a voxel of airspace and a voxel of tissue is coloured blue whereas larger distances are coded yellow. **Div-Dviii**) Boxplots showing quantitative comparisons between n = 6, independent COVID_S_, COVID_C_ and control, VOIs. Boxplots show data median - box centreline, interquartile range (25 -75 percentile) of data - box bounds, data range without outliers -whiskers, outliers were considered as values 1.5 times above or below the box bounds. Quantification of mean surface area to volume ratio, airspace connectivity, and mean septal thickness are shown respectively (** indicates p<0.001, * indicates p<0.05 and ~ indicates p=0.08) (calculated by one-way ANOVA two tailed with Tukey comparison, p-values and F statistic can be found in Supplementary Information). **Dix**) Shows the distribution of airspace diameters for all six VOIs in each group modal values for COVID_C_, COVID_S_ and Control = 8.9, 152 and 351 μm respectively.

To assess the utility of HiP-CT to detect lung changes in SARS-CoV-2-infected lungs, we imaged an intact upper right lung lobe acquired from the autopsy of a 54-year-old male patient who died from COVID-19-related ARDS (Figure 4A). The clinical features of this patient are described in the Online Methods. Orthogonal 2D slices through the volume of the lung imaged at 25 μm/voxel demonstrated high intensity regions in the lung periphery (see Figure 4Aii and Supplementary Video 5) consistent with patchy lung consolidation described by conventional clinical radiology in COVID-19^43^. Upon higher resolution scanning of VOIs at 6 μm/voxel, 2D slices depicted heterogeneity in the loss of normal alveolar architecture, with particular secondary pulmonary lobules displaying greater parenchymal deterioration than others (Figure 4Aiii). Higher magnification of the more affected secondary pulmonary lobule at 2 μm/voxel (Figure 4Avi) demonstrated cavitation of lung parenchyma, alveolar obstruction, (likely from thrombi based on their similar contrast to intravascular blood), thickening of septi between adjacent alveoli and blood capillary occlusion with adjacent cellular infiltrates (likely aggregations of lymphocytes ^41^) (Supplementary Video 5). These results indicate that HiP-CT is capable of reproducing the microstructural findings observed in COVID-19 lung biopsies^41,42^ across large tissue volumes, and in providing access to structures at novel length scales e.g. the secondary pulmonary lobule (Figure 4B).

Given the heterogeneity of parenchymal damage, we aimed to characterise changes of lung architecture in differing regions of the lobe with varying levels of parenchymal deterioration. We identified and segmented two adjacent secondary pulmonary lobules from the periphery of the COVID-19 sample (Figure 4B) which showed differing parenchymal deterioration termed COVIDS (less deteriorated) and COVID_C_ (more deteriorated). For the COVIDS lobule (yellow), we used the higher resolution 6 μm images to segment an acinus (Figure 4Cii) and compared this to an acinus segmented from the control lung (Figure 4Ci). These images show the loss of overall surface area in the lung and smaller and less uniformly shaped alveoli. To quantify this difference, we segmented the free air space using 2.5 μm/voxel scans in VOIs from COVID_C_ and COVID_S_ regions and compared these to an uninfected control lung (n=6 VOIs per group) (Figure 4Bi). 3D quantitative analysis was performed to assess microstructural changes, revealing a significant decrease in surface area to volume ratio (Figure 4Dvii) and increase in septal thickness (Figure 4Dviii) between control vs COVID_S_, COVID_S_ vs COVID_C_ and control vs COVID_C_ (p<0.001). Airspace connectivity is significantly higher for COVID_C_ than control (p=0.03) and significantly higher than COVID_S_ (p<0.001). These data (Figure 4Di–4Diii) and the distribution of airway diameters (Figure 4Dix), collectively quantify the heterogeneity of lung deterioration in SARS-CoV-2 infection. Thickening of alveoli septae leads to decreased airspace connectivity and a decrease in the modal airway diameter. In consolidated areas, infiltration of loose connective tissue and fluid into the alveoli leaves only tiny unconnected portions of the alveoli and the large well connected alveoli ducts ventilated. This is evident in the bimodal distribution of airway sizes and high airspace connectivity. Thus, HiP-CT detected regional changes in the architecture and morphology of the COVID-19 lung and allowed quantification, which can inform our understanding of the pathogenesis of COVID-19-related ARDS in SARS-CoV-2 infection.

## Discussion

Here, we developed HiP-CT, a novel phase-contrast based sCT modality utilising the first high-energy 4^th^ generation synchrotron source^23^. Through our various methodological developments, we have enabled hierarchical 3D imaging of multiple intact human organs, providing consistently high imaging quality from whole human organs down to individual organotypic functional units and certain specialised cells at any location within the organ.

Due to the mild sample preparation and non-destructive imaging, HiP-CT samples can be subsequently compared with other imaging techniques e.g. histology, to provide validation and aid in HiP-CT image labelling. Our technique has several key advantages over previous sCT based methods^1^: i) different resolution images from any location can be easily aligned with one another, facilitating data visualisation and interpretation; ii) whether a high resolution region is representative of the whole organ can be easily assessed; iii) data collection and storage are efficient, particularly for rare and or spatially distant features. Our exemplar of multiscale glomerular analysis demonstrates the benefit of hierarchical imaging with representative subsampling to estimate number and morphology of spatially disparate (glomeruli) organotypic features. We also leveraged the advantages of HiP-CT for multiscale and quantitative 3D analyses in pathological contexts, identifying and quantifying regional changes to the architecture of the air tissue interface and alveolar morphology in the lung of a donor with confirmed pulmonary COVID-19 disease.

Mapping the hierarchical structure of human organs is a major challenge in biology. International consortia to map the human body include the Human Cell Atlas and the Human BioMolecular Atlas Program^44,45^. Such consortia have generated data of the transcriptional states and cellular and molecular composition of human tissues^46^. To integrate HiP-CT into such efforts, a future challenge will be the validation and extension of automated cell detection and labelling. This may necessitate the development of contrast agents^47^ or of multi-modal imaging using techniques with established targeted labelling^3^. Another challenge is the computational power and storage required to handle the magnitude of data generated by HiP-CT. Whilst the hierarchical nature of HiP-CT is inherently data efficient, a single VOI through the depth of the lung captured at 6 μm/voxel still amasses ~600GB of data. Emerging cloud-based frameworks^48^ could improve the speed of image processing and analysis and facilitate the open accessibility of HiP-CT, providing a means to complement global efforts to map the human body.

HiP-CT has considerable translational potential for biomedical applications, which we demonstrated by 3D imaging a SARS-CoV-2-infected lung. In addition to reproducing the histopathological hallmarks of COVID-19, HiP-CT revealed extensive regional heterogeneity in parenchymal damage. Our HiP-CT approach clearly demonstrates how physical sub-sampling methods which aim to infer organ-wide pathophysiology could be confounded by such heterogeneity. This potential confounder is surmounted by HiP-CT due to its flexibility in the scale of tissue volume that can be imaged, independent of location and over a wide range of resolutions. Moreover, quantitative analysis of lung architecture from HiP-CT images align with clinicopathological observations of an increased volume of ventilated air that does not participate in gas exchange in COVID-19-related ARDS ^39,41^. Further HiP-CT investigation of COVID-19-related ARDS requires refinement using image analysis methodologies such as machine learning^49^. HiP-CT could also be used to provide insights into the secondary consequences of COVID-19 in other organs, such as the kidney and brain; both showing evidence of tropism for SARS-CoV-2^50^.

HiP-CT will evolve alongside advances in synchrotron technology. Here, we present the first results using our experimental method developed on a test beamline (BM05). The completion of a new beamline at the ESRF, BM18, in 2022 is anticipated to provide increased resolution, in volumes several times larger than human organs whilst using lower X-ray doses, with improved sensitivity and a much higher speed. Such 4^th^ generation synchrotron sources may herald new possibilities in the life sciences.

## Online Methods

### Sample details

Control organs were obtained from two bodies donated to the Laboratoire d’Anatomie des Alpes Françaises (LADAF). Dissections were performed respecting current French legislation for body donation. Body donation was based on free consent by the donors antemortem. All dissections respected the memory of the deceased. The post-mortem study was conducted according to QUACS (Quality Appraisal for Cadaveric Studies) scale recommendations^51^. COVID-19 lung samples were obtained from the Hannover Institute of Pathology at Medizinische Hochschule, Hannover (Ethics vote no. 9022_BO_K_2020). The transport and imaging protocols were approved by Health Research Authority and Integrated Research Application System (HRA & IRAS) (200429) and French Health Ministry.

Donor 1, from which the heart, lung, kidney, and spleen were imaged, was a 94-year-old, 45 kg, 140 cm female with right sylvian and right cerebellar stroke, cognitive disorders of vascular origin, depressive syndrome, atrial fibrillation and hypertensive heart disease, micro-crystalline arthritis (gout), right lung pneumopathy (3 years before death), cataract of the left eye, squamous cell carcinoma of the skin (left temporal region).

Donor 2, from which the brain was imaged, was a 69-year-old, 40 kg, 145 cm female, with type 2 diabetes, pelvic radiation to treat cancer of the uterus, right colectomy (benign lesion on histopathology), bilateral nephrostomy for acute obstructive renal failure, cystectomy, omentectomy and peritoneal carcinoma with occlusive syndrome.

Donor 3, from which the entire upper left lung lobe and a core biopsy from the periphery of the right upper lobe were obtained was a 54-year-old male patient who died from COVID-19, 21 days after hospitalisation. Treatment involved mechanical ventilation. Regarding comorbidities, arterial hypertension and type II diabetes were not diagnosed prior to death.

Donor 4, from which a biopsy from the right lung, lower lobe was taken was a male of 77 years. Resection of the lung segment was due to small pulmonary adenocarcinoma (1.4 cm), coronary heart disease, arterial hypertension, chronic rheumatic disease (polymyalgia rheumatica).

### Control Organ Autopsy and Organ Dissection (LADAF)

Bodies were embalmed with formalin solution as follows: embalming solutions - 4500 ml of formalin diluted to 1.15% in a solution containing lanolin and 4500 ml of formalin diluted to 1.44% were injected sequentially into the right carotid artery after death. Bodies were then stored at 3.6 °C. All eviscerations were performed in the LADAF, between April and July 2020.

During evisceration, vessels were exposed, and surrounding fat and connective tissue removed. Organs were post-fixed in 4% neutral-buffered formaldehyde at room temperature for one week. The lungs were inflated with the 4% formalin solution using a large syringe to inject the solution in the bronchia until a correct shape of the lungs was obtained.

### COVID Autopsy and Organ Dissection

All COVID-19 autopsies were carried out according to standard procedures by the Deutsche Gesellschaft für Pathologie and followed regulatory requirements. Autopsies were performed in a room with adequate airflow (>6 air changes per hour of total room volume) using appropriate personal protective equipment (i.e., hazmat suits, boots, goggles, FFP2/3 masks). Organs were eviscerated and immediately fixed in 4% neutral-buffered formaldehyde. Lungs were immediately inflated and fixed by tracheal instillation with 4% neutral-buffered formaldehyde, the trachea was then clamped, and specimens were left for post-fixation in 4% neutral-buffered formaldehyde at room temperature for ≥72 hours before further dissection. Samples were partially dehydrated to 70% ethanol (through four successive concentrations see below) prior to transportation to ESRF. COVID-19 lung samples were transported to ESRF, Grenoble at ambient temperature in double-sealed containers.

### Organs preservation and degassing

To ensure resistance of the samples to the X-ray dose, avoid health risks associated with formalin; all organs were equilibrated in a 70% ethanol solution. In addition, ethanol replaces water in tissues, thus the density of the water-rich tissues and water-filled cavities decreases, while the density of other tissues remains closer to the original one. This improves X-ray contrast.

To avoid organ shrinkage, serial dehydration was performed through: 50%, 60%, 75%, and finally, 70%. Each solution was 4x volume of the organ. The time at each concentration is dependent on organ size and tissue composition (adipose tissues requires longer). In all cases adequate dehydration can be assessed by tapping the organ container and looking for streaks in the solution. The slowest organ to prepare is the brain owing to its size and high adipose content. In our case, brain was dehydrated over 3 weeks, however optimisation of this step has not been performed and it is likely that this can be done more quickly if required^8^.

Degassing was performed at each dehydration step with the organ immersed in the respective solution using a diaphragm vacuum pump (Vacuubrand, MV2, 1.9m^3^/h), to remove both free and dissolved tissue gas.

Degassing cycles were performed with increasing duration from 2 minutes to 40 minutes (total time typically 2h). At each cycle, pumping was stopped when bubbling decreased in intensity, or became intense enough to potentially cause organ damage. For Donor 1 lung, after each cycle, the degassed ethanol solution was gently forced into the main conducting air ways with a large syringe in order to keep the morphology of the lung in its original inflated state.

The final degassing step, was longer (typically 3-4 hours), performed until no bubbling was observed. Samples were then mounted. It should be noted that some damage attributed to degassing was observed in the brain of Donor 2 and the protocol was amended to use a series of thermal cycles.

The brain is immersed in a bath of pre-degassed appropriate concentration Ethanol, this is stored at 4°C for 5 days to equilibrate. 5 cycles with successively increasing ethanol concentration are used (50%, 60%, 70%, 70%, 70%). This protocol requires vacuum degassing only for the final mounting of the organ, however it requires ~5 weeks. It may be possible decrease the time by combining gentle vacuum degassing, with thermal cycling.

### Organ mounting with Agar-agar ethanol gel preparation

Organ mounting requires delicacy, as any remaining gas bubbles, density inhomogeneities or insufficient sample stabilization dramatically reduces scan quality. The 70% ethanol-based mounting medium was prepared using demineralized water (20 g/L of agar). Once gelled, the block was cut into small cubes of 1 cm^3^ that were immersed in ethanol at 96%, the volume ratio ensuring an equilibrium conc. of 70% of ethanol (2.96 L of ethanol at 96% for 1 L of gel) for ~24 hrs. Equilibrium was assessed by the gel cubes sinking (over several dozen seconds) to the bottom of the container after agitation. Once equilibrated, the gel cubes still immersed in the solution were degassed for two cycles of approximately one hour each, until no bubbles formed.

A third of the cubes were stored in the in this degassed equilibrated state, the remainder were blended or more recently crushed into “blended gel”, the consistency of which was adjusted by adding 70% ethanol, or removing the ethanol by pressing with a filter.

### Organs mounting and degassing

During the exploratory phase of this project, several mounting protocols were tested. The first method, used for the COVID-19 lung-lobe consisted of using only the agarose gel cubes. This preparation allowed efficient final degassing but caused unwanted local compression of the soft organs (visible in Supplementary Video 5).

The final protocol developed, filled the bottom of the cylindrical container with gel cubes in a few centimetres of blended gel, to ensure the organ does not contact the container base and preventing rotation of the sample. The container was then half-filled with blended/crushed gel, and the organ carefully immersed and set in the desired position (with care to avoid bubbles). The organ was covered completely with blended/crushed gel. The whole container was then degassed, with care to ensure that the gel did not inflate too much due to trapped gas bubbles. Once degassed for 1h (or few minutes for brains), the mounted organ was covered with gel cubes to ensure solid fixation. The whole mounting was degassed a final time for few minutes, then the container was sealed for scanning.

### Specific sample holder and mounting

We used specially designed sample holders for the scanning process. They were designed to ensure safety in case of a leak of the ethanol via a double sealing, to be mechanically stable and resistant to high X-ray dose. Two samples can be mounted one over the other for longer scan automation. Alternatively, only one organ can be installed, and the equivalent organ container filled with 70% ethanol solution is installed in the second place for beam reference measurements in case of local tomography scans (Figure 1C).

### Beam properties and Hutch design

The ESRF-EBS provides a coherence length of 8 μm at 70 keV on BM05. The polychromatic beam provides a high flux whilst enabling a longer propagation distance before holographic imaging mode is reached. Both these features allow longer propagation distances before reaching the geometric blurring limit due to the angular source size up to the near-field limit, or a bit beyond. On BM05 the propagation distance is physically limited to 3.5 m. For both 6.5 and 25 μm/voxel scans, this restriction prevents us reaching the theoretically estimated maximum propagation distance limit at 70 keV (respectively 6 m and 23 m) defined by the minimum between the geometric blurring of 1 pixel and the near-field limit, whilst for 2.5 μm/voxel and lower, the propagation distance limit is 1.3 m (estimated to be close to the near field limit).

The beam flux and energy must be tuned for each magnification and sample to ensure the average energy is sufficient to penetrate through the sample but low enough to allow fast scans. Tuning is achieved with a combination molybdenum and copper filters (energy tuning) and fused silica bar attenuators (flux and beam profile tuning). To preserve the beam coherence the filters and optics are made of high-quality mirror polished materials to ensure material homogeneity. Similarly the X-ray optics are made of pf6/If1 beryllium which can be highly polished. The scintillators must be as dense as possible whilst not degrading the resolution. LuAG:Ce provide the necessary stopping power with a high light output and the transparency required for high resolution phase contrast. The scintillators thickness was optimised to provide a compromise between light output and maximum optical resolution for each X-ray optic.

All scans were taken using one of two detector optics: the d-zoom (for 25-6.5 μm/voxel scans) and the zoom optic for 6-1.3 μm/voxel scan**s**. Both optics were mounted with PCO edge 4.2 CLHS cameras. In designing the optics we aimed to have the highest possible numerical aperture without having to reduce thickness of the scintillator and hence reduce resolution. The greatest limitation for the detector optics, particularly the zoom optic, is darkening due to the polychromatic beam. To prevent darkening the optic is either intrinsically X-ray hardened (as is the case for the d-zoom optic) or is protected from darkening through the use of thin glassy carbon mirror to reduce internal scattering, and lead glass in the front of the optic to stop as much scattering as possible. A beam stop was also used to prevent beam back scatter. Finally, we developed a rapid curing process using blue LEDs to recover optics in 12 hrs in the case that optics did darken. 2048x2048 pixel scientific Complementary Metal Oxide Semiconductor (sCMOS) detectors were chosen for HiP-CT to avoid data loss during read out time and benefit from their 74% quantum efficiency at the peak emission of the LuAG:Ce. The lower full well capacity of the sCMOS by comparison to CCD detectors is efficiently mitigated through our accumulation scanning protocol.

### Multi-resolution scanning protocol

One organ in its sealed container was installed in the bottom holder, and the equivalent container filled with 70% ethanol gel was installed in the upper holder (Figure 1C). This second container was used for acquisition of beam references. Partial angular integration of scans from the reference jar every 100 projections provided a new reference for flat field correction of the radiographs. In this way, most of the low frequency effects of local tomography were directly corrected, leading to normalized intensities of the projections even in case of off-center local tomography scans (see Figure 1B).

This approach also improves the dynamic range of the detector by avoiding direct exposure to the beam- the sample and its mounting media act as filters. This protocol, we term the “attenuation protocol”, is derived from an approach that was originally developed to scan highly absorbing fossils, first in monochromatic beam^24^, and later in polychromatic beam^25^. In this protocol, the dynamic level was maximised for scanning organs at 25 μm/voxel, by putting the sample slightly off axis to benefit from the natural horizontal gaussian profile of the beam. This approach, reduces the flux on the border of the sample (where the absorption is lower), and provides maximum flux to the thickest portion of the sample.

As mentioned above, in addition to correction of the local tomography effect, our scanning protocol avoids direct exposure of the detector to the beam, allowing the saturation level of the detector to be adapted to the less absorbing part of the sample instead of to the direct beam intensity. As the general absorption contrast is dominated by the cylindrical shape and the organs are close in density to the ethanol solution, this approach also efficiently removes any beam hardening effect, or differential phase contrast effect in case of off-axis scans. Finally, in order to ensure a high dynamic level, the detector is used in accumulation^26^, with typically 10 and 5 sub-frames for the 25 μm/voxel and 6.5 μm/voxel scans respectively. The 2.5 μm/voxel and 1.3 μm/voxel scans are done without accumulation to limit the X-ray dose and because their contrast is dominated by phase-contrast. As phase-contrast is typically 1000 times more sensitive than absorption, it is possible to use lower dynamic level on the detector, and thus reduce the X-ray dose. The benefit of each aspect of this scanning protocol is demonstrated in Extended Data 6

Two acquisition modes were used depending on the size of the organ samples – half and quarter acquisitions. Most scans were performed in half-acquisition mode with the centre of rotation on the right side of the field of view (moved by typically 900 pixels) to obtain a field of view of 3800 pixels with 6000 projections. For the largest organs, the complete scans at 25 μm/voxel were performed using a quarter acquisition protocol based on two scans (one half-acquisition + one annular scan) of 9990 projections each. Concatenation of the quarter acquisition was performed by calibrating the overlapping scan region with a gradual transition and a normalisation of the grey levels in the common area of the two scans. Once concatenated, the reconstructed field of view is 6000 pixels. To cover complete organs or to scan large columns in local tomography, an automated z-axis series was performed. The typical sampling step was 2.2 mm vertically for a corresponding beam size of 2.6 mm corresponding to an overlap of 18% (some initial organ scans used larger steps (up to 3.6 mm), and larger overlap - upto 50%).

All scanning parameters are presented in Table 2 and Supplementary Data 1. Based on the scan times possible (Extended Data 1) the brain supplied by Donor 2 can be scanned at full 25 μm resolution in ~16 hrs (16 cm); and the smallest diameter organ – the kidney (7 cm) in ~3.5 hrs.

### Tomographic data reconstruction protocol

Prior to reconstruction 25 μm/voxel radiographs were vertically concatenated and the remaining vertical profile of the beam (regular horizontal lines) subtracted (at 6 μm/voxel and lower this step is unnecessary as the scans are not attenuation dominated). For higher resolution scans we first performed tomographic reconstruction, then cross correlation between slices to ensure alignment. For overlapping slices we use a ponderate average to create a gradual transition from one slice to the next. This removed horizontal artefacts in these phase-dominated scans. After pre-processing, tomographic reconstruction was performed using the filtered back-projection algorithm, coupled with a single distance phase retrieval^27^, and a 2D unsharp mask on the projections, as implemented in the ESRF inhouse software PyHST2^28^. All sub-volumes were converted into 16 bits, and vertically concatenated. The remaining ring artefacts were corrected on the reconstructed slices using an inhouse Matlab system derived from Lyckegaard et al.^29^. In some cases, a final correction of the horizontal stripes was performed on the reconstructed volumes after vertical reslicing.

### Histology (haematoxylin and Eosin)

After HiP-CT scanning the kidney from Donor 1 was removed from the mounting jar section and stained for histological validation (N=1). The coronal location of the kidney aligned with a HiP-CT high resolution image column was manually identified and the kidney dissected. Large coronal sections were placed into cassettes to follow the standard tissue treatment procedure: dehydration through a series of graded ethanol baths, paraffin embedding and microtome sectioning of paraffin blocks. Slides were routinely stained with haematoxylin and eosin (H&E). Slides were visually inspected to identify precisely aligned images to high resolution HiP-CT.

In addition to performing H&E staining on the HiP-CT imaged kidney in the portions of the sample exposed to the highest X-ray dose (Figure 3B and Extended Data 5), staining was also performed on the COVID-19 lung biopsy sample (N=1) shown in Figure 1E and on healthy control (Donor 4) lung biopsies. (Extended Data 7) shows H&E staining as well as anti-CD31, anti-thyroid transcription factor 1 (TTF-1) and anti-fibrin immunohistochemical staining on the lung biopsy samples. As these lung samples were biopsies, they did not receive the high radiation doses the kidney was subjected to (see Supplementary information for discussion and estimation of dose exposure), however these sample underwent similar preparation and were exposed to the ESRF-EBS on BM05.

### Image Analysis and Statistics

The final volume renderings were performed using different 3D software packages, notably VGStudioMax 3.2.4 (Volume Graphics, Heidelberg, Germany), Amira v2019.6 and Dragonfly 2020.2.0, For all analysis ImageJ version 2.1.0/1.53c Java 1.8.0_66 was used.

### Fourier Shell Correlation (FSC)

FSC was performed on the Donor 1 Lung sample. Two independent scans taken sequentially (after 0.1 mm of vertical displacement of the sample and of the center of rotation) and using the full number of projections for both were fully reconstructed as per the normal HiP-CT procedure with the complete chain of pre- and post-processing. The pairs of volumes were loaded in VGStudioMax 3.2.4 and precisely aligned. The common part was then cropped and exported in the same 16 bits tif format, resulting into two volumes of same size and same location, but with fully independent acquisition and reconstruction. 9 randomly selected subvolumes of data at sizes ranging from 200 - 1000 voxels were taken from both aligned volumes and the FSC computed for each. The ½ bit threshold intersection with the FSC curve was used to estimate the resolution corresponding to each voxel size. The mean and standard deviation of the intercepts is provided and the FSC curves are provided in Extended Data 3.

### Image quality comparison (Figure 1B, Dii and Diii)

SNR ratio was evaluated from the mean (μ) and standard deviation (σ) of pixel values in manually drawn ROIs of features (ft) and background (bg) areas. The SNR was calculated as (μ_ft_ -μ _bg_)/σ _bg_. In the kidney the tubule walls were used as the features with the lumen of the same tubule used as the background region. N=3 tubules per lateral resolution image were analysed.

Image quality comparison was performed using the structural similarity index^30^ implemented in Matlab 2020b (ssim function). Equally sized subvolumes from the two columns were used in their original 32-bit form (999 slices in each case). Histograms of the two subvolumes were calculated with fixed bin width of 0.001. Skew, kurtosis and mean pixel intensity were calculated via Pyradiomics v3.0.^52^. Structural similarity index was calculated between pairs of xy slices from the two subvolumes. Two images were randomly selected (by slice number); either both images were from the same subvolume (1-1) and (2-2) or the images were from different subvolumes (1-2) and (2-1). N=200 pairs of images were analysed for each group. One-way ANOVA of the output was performed in Matlab 2020b using the anova1 function.

### Kidney Glomerulus analysis

Using the 25 μm/voxel scans of the whole kidney, the parenchyma of N=1 kidney was semiautomatically segmented in Amirav2019.6. After manually aligning all three resolution datasets, two cylinders, one through each of the 6 μm/voxel and 1.3 μm/voxel columns, were defined perpendicular to the kidney surface. The cylinders had radii, lengths, and volumes of (1300 μm, 40,811 μm and 2.17x10^11^ μm^3^) and (203 μm, 9814 μm and 1.3×10^9^ μm^3^) for the 6 μm/voxel and 1.3 μm/voxel datasets respectively. These cylinders were considered as virtual biopsies. In the 6 μm/voxel case any glomeruli that fell within the cylinder were counted (blue dots in Figure 3Aiii). For the total number of glomeruli, the volume of parenchyma in the 6 μm/voxel virtual biopsy was found via semi-manual segmentation in Dragonfly 2020.2.0. The number of glomeruli counted in the 6 μm/voxel virtual biopsy was divided by the volume of parenchyma in the 6 μm/voxel virtual biopsy and multiplied by the total volume of the parenchyma. In the 1.3 μm/voxel dataset any glomeruli that touched the cylinder were manually segmented in Amira v2019.6 in 3D. The volume, surface area and sphericity were calculated using the binary mask from these segmentations in ImageJ using the MorphoLibJ plugin-(analyse regions 3D)^53,54^. Alignment of the HiP-CT and H&E histology sections was performed in VGstudioMax 3.2.4. Pseudo coloring of the HiP-CT was performed by applying a 100 pixel median filter on the histological slice and superimposing these colors to the original grey level slice of the HiP-CT. Grey-level version of the histological slice was obtained by desaturating the picture, inverting its contrast, and adjusting linearly the contrast to reach 0.2% of saturation in black and white on an 8 bit scale.

### Lung microstructure analysis

Manual segmentation of secondary pulmonary lobules was performed in Amira 2019.6 using the 25 μm/voxel COVID-19 upper right lung lobe scans. The data was binned to 50 μm/voxel prior to segmentation to reduce computational load. Acini segmentation was performed using column of 6 μm/voxel data in the control lung and a region of the COVID-19 lung selected by pulmonary radiologist as relatively spared. Regions of the image that contained a clear terminal bronchiole were cropped and segmentation was performed semi-manually: first tissue air boundaries were enhanced using an unsharp mask and a Canny edge filter in ImageJ^53^, enhanced images were then transferred to Aimia2019.6 and a 3D magic wand tool was used segment the 3D airspace. For microstructural analysis, N = 6 250×250×250 pxl subvolumes from the 2.2 μm/voxel datasets of the COVID-19 and control lung, were chosen, (randomly in the case of the control lung, and in the COVID-19 case, randomly within larger areas designated by pulmonary histopathologists as structurally preserved or consolidated i.e. from areas where alveoli morphology was identifiable versus areas where there was widespread consolidation of the parenchyma). Binary images separating airspace from alveolar septae and microvessels were produced in ImageJ by thresholding the images using one of the two following methods: ‘triangle’ method for Control and COVID_S_ and ‘Yen’ method for COVID_C_^53^. A 3D Chamfer distance from the MorpholibJ plugin ^54^ was used (using Svensson 3,4,5,7) to create the distance maps (Figure 4Di-iii). The BoneJ plugin (V2) was used to perform morphological analysis including connectivity, surface area to volume ratio, airway diameter and septal thickness^55,56^. One-way ANOVA with Tukey comparison was used for statistical analysis of microstructural measures.

## 1 Extended Data

**Extended Data Fig. 1:**
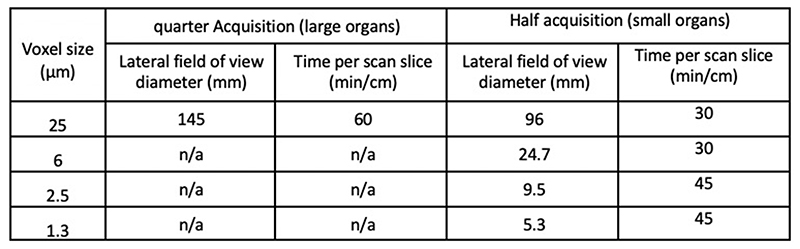
Minimum scan times Minimum scan times for quarter and half acquisition modes at voxel sizes presented in this work.

**Extended Data Fig. 2:**
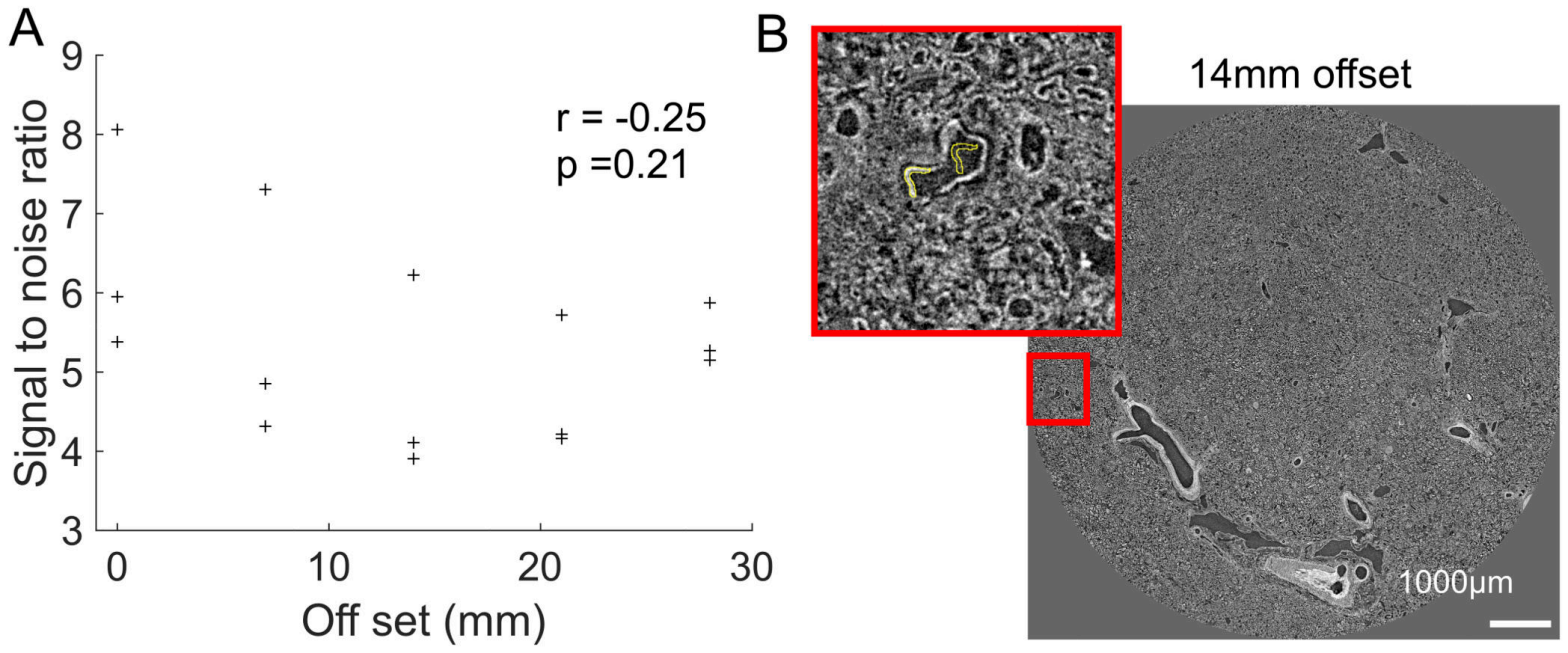
Signal to noise variation with lateral distance from center of rotation Graph showing the signal to noise variation with lateral off-set from centre of rotation. Pearson correlation coefficient (r) = -0.25 and p-value =0.21 (two-tailed test) shown on graph. It should be noted that the exposure time of the camera and final selection of the X-ray filters were set-up on the most off-centre scan, at the angle where the beam was crossing the smallest amount of material, in order to ensure that detector saturation would not occur during scanning. B) showing an example of the regions used to calculate the SNR. Signal was taken from tubule walls while the open tubule lumen was used as the background region.

**Extended Data Fig. 3:**
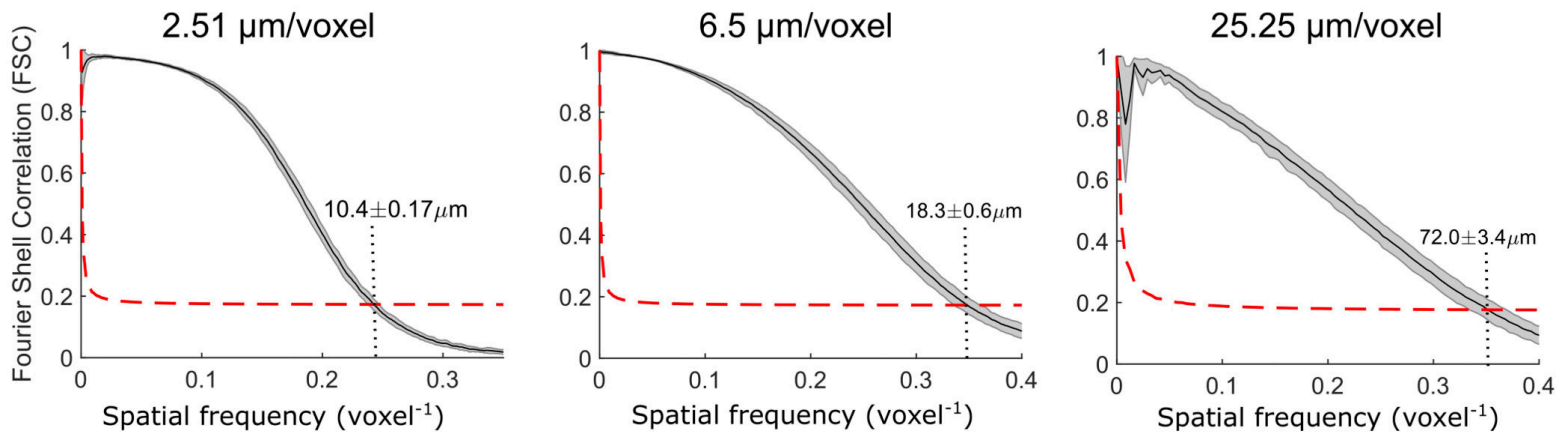
Resolution estimation via Fourier shell correlation Resolution estimation from fourier shell correlation (FSC) measure. FSC curves (black) with standard deviation (grey shaded) and ½ bit criterion (red hatched) for each voxel size imaged Donor 1 lung. Intercept point is marked and labelled with the equivalent spatial resolution. FSC was calculated in each case using 9 subvolumes cropped at randomly varying spatial locations from the total image volume. Subvolume sizes were varied (200,500 and 1000 voxels).

**Extended Data Fig. 4:**
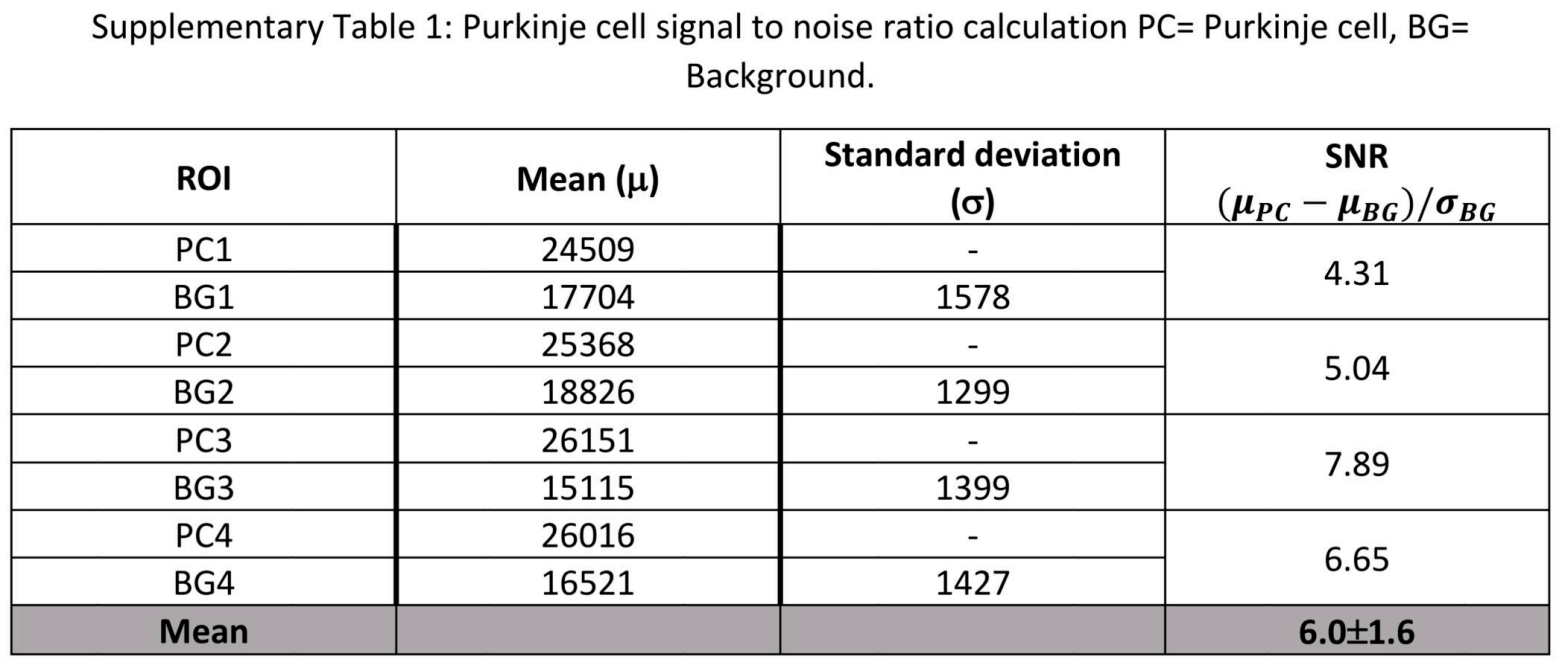
Purkinje cell signal to noise ratio Purkinje cell signal to noise ratio calculation PC= Purkinje cell, BG= Background.

**Extended Data Fig. 5:**
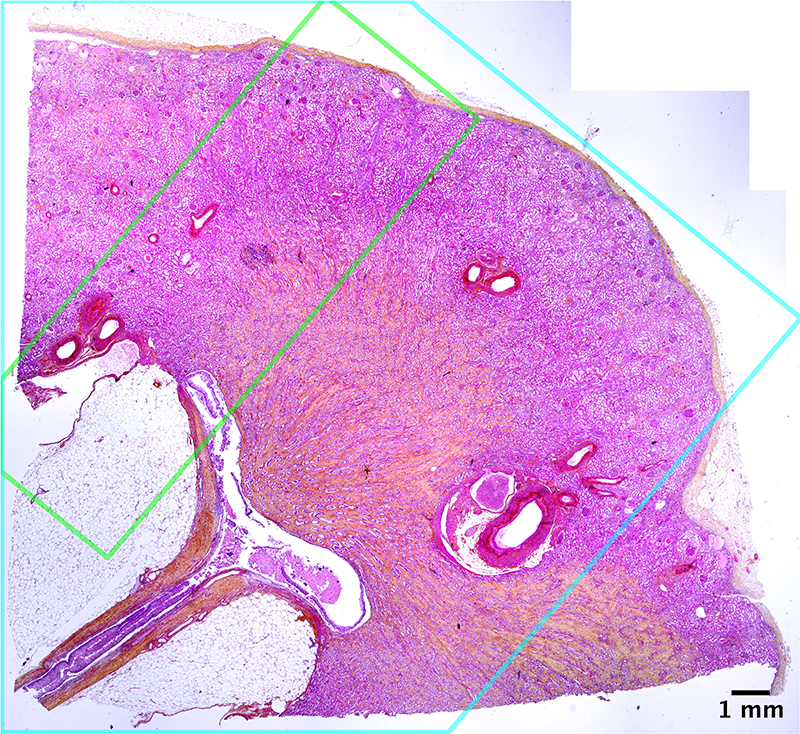
High X-ray dose does not cause detectable damage to kidney as assessed standard histology H&E Large scale view of Haemotoxylin and Eosin (H&E) stained slice of the kidney with outlines of the 6 μm/voxel (blue) and 1.3 μm/voxel (green) scanning regions shown. There is no visible difference to suggest X-ray damage to parenchyma between these regions (N = 1).

**Extended Data Fig. 6:**
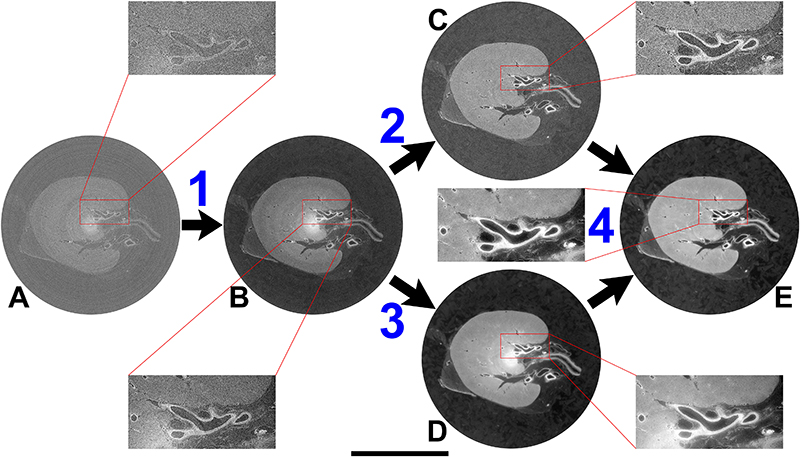
Imaging protocol developments The attenuation scanning protocol was originally developed to image dense fossils and adapted for HiP-CT of complete human organs. In this protocol, the centre of rotation is in the middle of the cylinder used to mount the sample. There are four key principles: 1) single distance phase retrieval using Paganin et al. algorithm coupled with an unsharp mask on the radiographs after phase retrieval; 2) optimization of references absorption profile for local tomography; 3) optimization of dynamical range by adjusting detector saturation level through the sample; 4) a combination of 1, 2, 3 and the attenuation protocol adapted to organs. **A**) classical scan in edge detection mode. The saturation level of the detector is set on the beam reference without the sample **B**) Same scan reconstructed using single distance phase retrieval. **C**) Same scan but references are calculated from an equivalent jar filled with mounting media instead of using pictures of the beam without sample. **D**) Scan performed off-axis to have a lateral gradient of power in the beam to fit with absorption profile of the sample. Saturation level of the detector tuned through the sample, normal references without sample in the beam. **E**) Scan in attenuation protocol using references in equivalent jar filled with mounting media, dynamic range optimization and single distance phase retrieval. Scalebar 50mm

**Extended Data Fig. 7:**
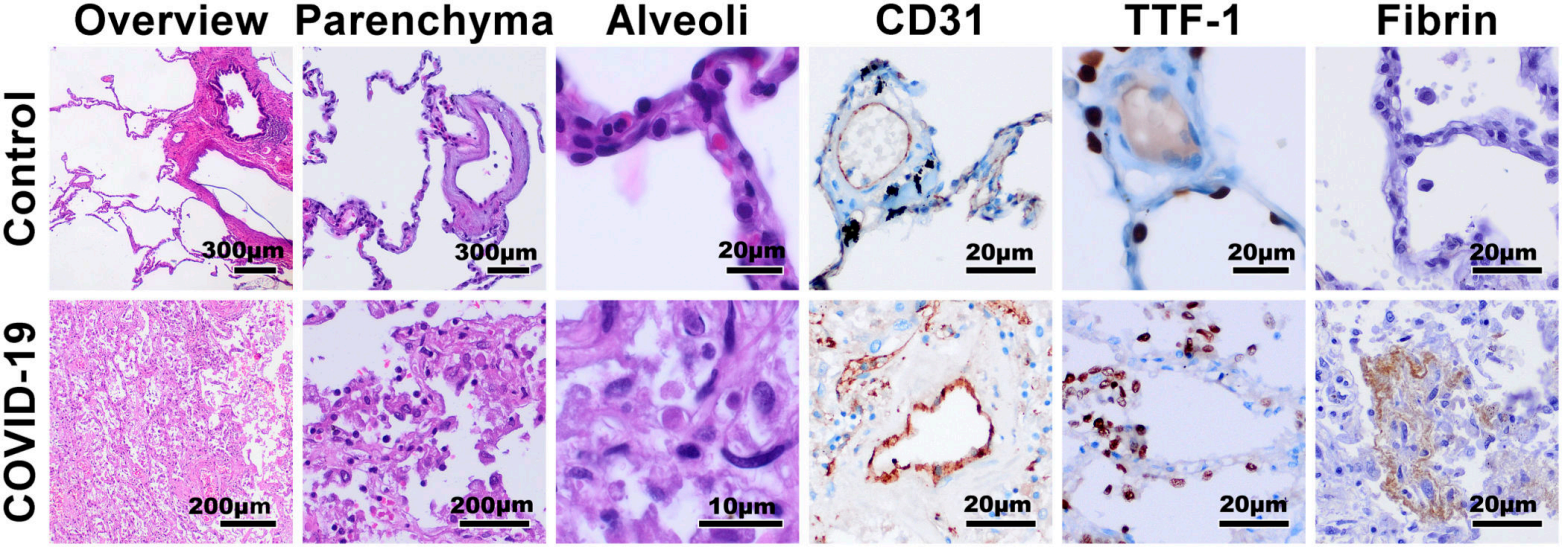
Histological staining following imaging of lung tissue of BM05 H&E and IHC staining of COVID-19 lung biopsy and control (Donor 4) lung biopsy after HiP-CT imaging. H&E staining of overview, parenchyma and alveoli in columns 1-3; CD31 (column 4), Thyroid transcription factor 1 (TTF-1 (column 5)) and Fibrin staining (column 6) (N=1). For histological comparison, representative lung samples were cut and embedded in paraffin. 2 μm thick sections were cut followed by histological staining using Hematoxylin and Eosin (HE) at the Institute of Pathology at Hannover Medical School. Immunohistochemistry for CD31 1:75 (M0823, Agilent Dako, California, USA), TTF-1 RTU (790-4756, Hoffmann-La Roche, Basel, Swiss) and Fibrin 1:500 (MABS2155, Merck Millipore, Massachusetts, USA) were stained on a VENTANA BenchMark ULTRA (Hoffmann-La Roche, Basel, Swiss) with the aforementioned dilutions and pretreatment and incubation times according to the manufacturers advice. Representative images were acquired with a Olympus CS50 camera (Olympus, Tokyo, Japan) using Olympus cellSens Software (Olympus, Tokyo, Japan) on a routine diagnostic light microscope (BX43, Olympus, Tokyo, Japan).

## Supplementary Material

Source_data_Figure_1

Source_data_Figure_3

Source_data_Figure_4_Dvi-Dvii

Source_Figure_4_Dix

Supplementary Data 1

Supplementary Information

Supplementary Video 1

Supplementary Video 2

Supplementary Video 2

Supplementary Video 4

Supplementary Video 5

## Figures and Tables

**Table 1:** Overview of the techniques and capabilities developed and utilised to enable HiP-CT

Source and beam	E - 4th generation high energy storage ring with decreased source size / increased spatial coherence		I - polychromatic beam for simplified phase contrast approach and high flux
Filters / optics	E - **Use of high purity / highly polished windows and filters to preserved spatial coherence**		I - Metallic filters and fused silica bars to allow tuning of flux, beam profile and energy
Optimised scan parameters	NC- **Fine tuning of beam properties to balance transmission through sample against scan speed**		E- **Longest possible propagation distance until the limit of geometric blurring (or physical hutch limit**)
Scanning geometry	E - **Exploiting the horizontal beam profile, to adapt to the attenuation profile of the sample at low resolution**.		E- **Performing scans in an equivalent jar with only mounting media, to generate adaptive flatfield references to normalize the absorption profile**
Scintillator	I - **Using high light output, high atomic number, high density crystal scintillator for optimal quality at high energy: LuAG:Ce**		NC - **Optimising the scintillator thickness for each magnification to find the best compromise between pure resolution and signal level**
Detector optics	NC - **Using optics with the highest possible NA to optimize light**		NC - **Making detectors radiation hard to limit the darkening of the optics by use of lead glass and**
	**collection efficiency. Matching this to the scintillator thickness**		**glassy carbon mirrors. Rapid restoration process for darkened detectors using blue LEDs**
Detector sensors	NC - **2048x2048 sCMOS with small pixels used to ensure rapid readout, high quantum efficiency, low electronic noise**.		I - Recovery of large full well capacity through the use of frame accumulation when required (at lower resolution when attenuation contrast is dominant)
E- Essential	I - Important	NC- Non-critical	**Specifically developed/optimised for HiP-CT**

**Table 2:** Scan Parameters for all samples

Sample	Voxel size (μm)	Data label	Average energy of the incoming beam (keV)	Lateral Field of view (diameter (mm)	Scan time (hrs)
Donor 1 heart	25.08 μm	Complete organ	~85 keV	96	18
	6.05 μm	Left and right ventricle muscle + ramus interventricularis anterior	~87 keV	24.7	6
	2.22 μm	Left ventricle muscle	~76 keV	9.5	4
Donor 1 left lung	25.08 μm	Complete organ	~85 keV	145	24
	25.25 μm	FSC A&B	~80 keV	145	3
	6.05 μm	VOI-06	~81 keV	23	4
	6.5 μm	FSC A&B	~80 keV	24.7	2
	2.45 μm	VOI-02	~70 keV	9.5	3
	2.45 μm	VOI-06	~70 keV	9.5	3
	2.51 μm	FSC A&B	~70 keV	9.6	2
Donor 1 left kidney	25.08 μm	Complete organ	~85 keV	85	2
	6.05 μm	Central column	~85 keV	24.7	2
	1.29 μm	Central column	~69 keV	5.3	3
Donor 1 spleen	25.08 μm	Complete organ	~85 keV	85	2
	6.05 μm	Central column	~85 keV	24.7	1
	1.29 μm	Central column	~69 keV	5.4	2
Donor 2 brain	25.08 μm	Complete organ	~85 keV	145	22
	6.05 μm	Cerebellum – occipital lobe	~81 keV	24.7	5
	2.45 μm	Cerebellum	~74 keV	9.5	5
Donor 2 kidney	25 μm	Complete organ	~84 keV	85	5
	2.5 μm	Lateral transect	~80 keV	9.5	1
Donor 3 upper lobe of left lung	26.38 μm	Complete upper lobe	~66 keV	96	11
	6.24 μm	VOI-4		24.7	24
	2.22 μm	VOI-1.2b	~64 keV	24.7	16
	2.22 μm	VOI-8.2	~76 keV	9.5	7
Donor 3 upper lobe of right lung	2.25 μm	Core biopsy	~43 keV	9.5	3
Donor 4 lower lobe of right lung	2.25um	Core biopsy	~78 keV	9.5	2

## Data Availability

Image data that support the findings of this study are publicly available (with sign in) from the ESRF data repository. human-organ-atlas.esrf.eu Individual DOIs for all datasets are listed in Supplementary Table 2

## Software and Code Availability Statement

HiP-CT data were reconstructed using custom code written in Matlab2017 available at: https://github.com/HiPCTProject/Tomo_Recon and the PyHST2 software package (https://software.pan-data.eu/software/74/pyhst2) Volume renderings were performed using 3D software packages, notably VGStudioMax 3.2.4 (Volume Graphics, Heidelberg, Germany), Amira v2019.6 and Dragonfly 2020.2.0

Image quality comparison was performed using Matlab 2020b (ssim function). ImageJ v2.1.0/1.53c Java 1.8.0_66 and pecific plugins - BoneJ v7.0.11 and MorpholibJ v1.4.2.1 were used for lung morphology analysis, pyradiomicsv3.0 (https://pyradiomics.readthedocs.io/en/latest/index.html) was used to calculate skew and kurtosis of image histograms. FSC analysis code was slightly modified from https://github.com/bionanoimaging/cellSTORM-MATLAB/blob/master/Functions/FSC.m
