# Supplementary material for: Imaging intact human organs with local resolution of cellular structures using hierarchical phase-contrast tomography
Source: Nat Methods. 2021 Nov 4;18(12):1532–41. doi: 10.1038/s41592-021-01317-x (PMC8648561; doi:10.1038/s41592-021-01317-x)
Supplement: Supplementary file 1 [file 41592_2021_1317_MOESM1_ESM.pdf]

nature portfolio

https://doi.org/10.1038/s41592-021-01317-x

Supplementary information

Imaging intact human organs with local resolution of cellular structures using hierarchical phase-contrast tomography

In the format provided by the authors and unedited

Supplementary Information

1. Beam configuration

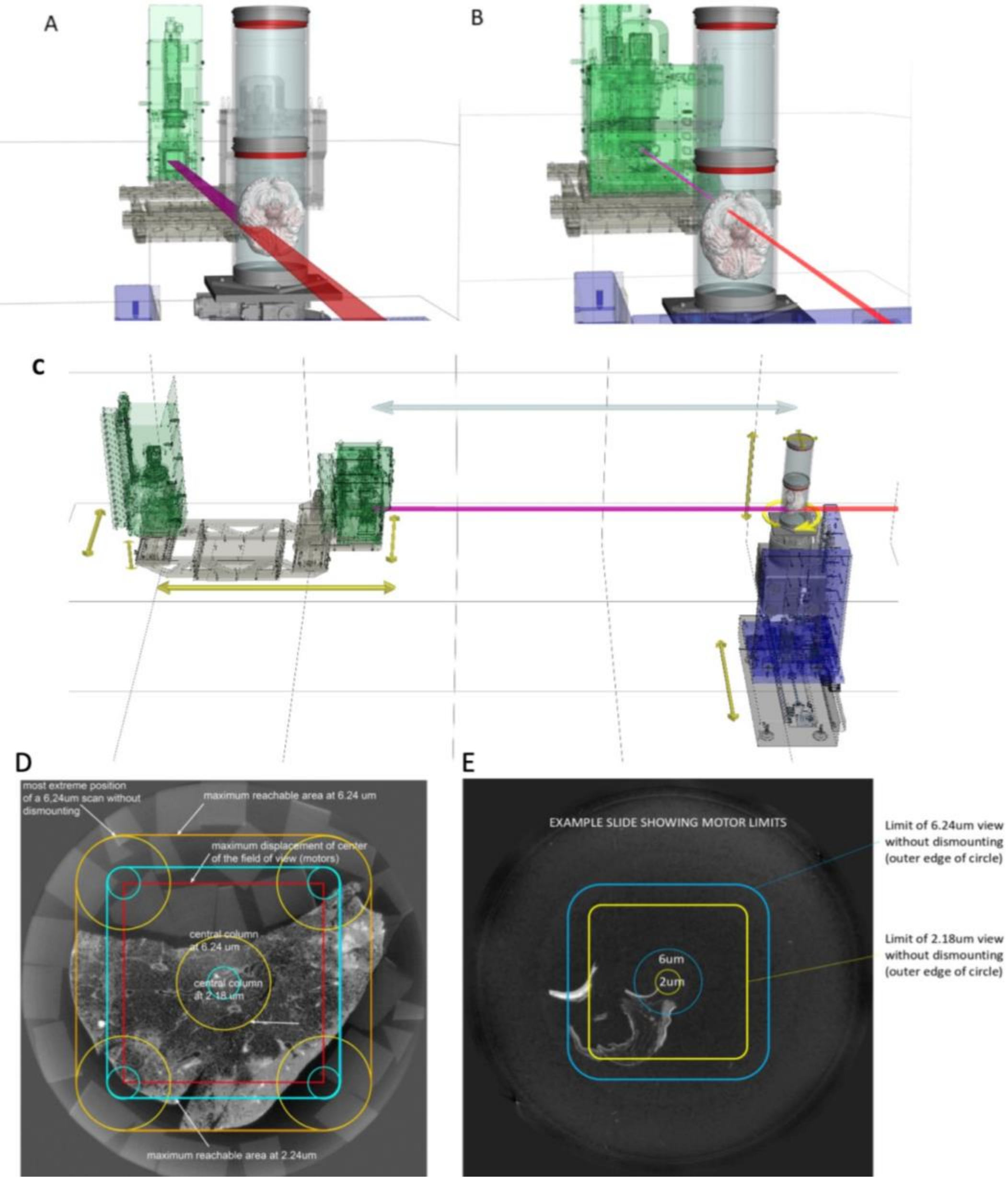

Supplementary Figure 1: Beam line configuration of human brain sample. Two containers (upper reference container and lower container holding the human brain sample) and the beam (red/magenta) are displayed in both images. A) 25 µm per voxel whole organ scan using the dzoom optic (green structure). B) a 2.5µm or lower resolution scan using the zoom optic (green structure). C) Experimental configuration of BM05, yellow arrows denote possible stage movements, and grey arrow shows propagation distance. The red line indicates the beam. The two optics -the two green structures are on the left and the sample stage with sample jar can be seen on the right. (D) and (E)Motor limits and VOI selection in the half and quarter acquisition modes respectively.

2. X-ray dose calculations

The X-ray dose delivered to the surface of the sample with HiP-CT is much higher than for biopsied tissue and is estimated (as water equivalent surface dose) through a series of measurements with a dosimeter that have been used to develop a dose rate estimator for any beam configuration used on BM05, as shown in (**Supplementary Data 1**). The dose integrated on a small volume of interest in local tomography is lower than the total delivered dose, as a large part of the incoming X-rays is absorbed in the surrounding tissues and mounting media. Nevertheless, for comparable signal level, the dose will always be higher in HiP-CT than when scanning core biopsies, because the signal has to cross a lot of material before reaching the detector. The necessary total delivered dose to the sample to reach similar level of quality in HiP-CT compared to core biopsies is then about 4 times higher, for integrated dose in the VOI of typically twice higher. The main consideration for dose with ex vivo samples is to prevent bubble formation, which causes artefact and prevents ridged registration. These bubbles appear when a given level of integrated dose is reached. Once started, each new scan will increase the problem, even if done in a different location. the only way to recover the sample and make it suitable for new scans is to perform a new vacuum degassing. The tolerance of the sample to high level of dose is extremely dependant of the initial degassing level. During the early phase of this project, it appeared that careful degassing makes the sample able to handle total dose at least 10 times higher than without degassing. After a series of scans, putting the sample at 5 degrees for typically two days if no bubbling occurred helps a lot to prevent bubbling at the next scanning session. Tissue damage may also be caused by excessive X-ray dose. At present, we have shown that even at the highest dosed areas (1.3µm columns that fall entirely within 6µm columns) histology with standard H&E can be performed and shows no obvious morphological damage (**Figure 3C**). Further evidence is shown in **Extended Data 5** where the H&E stained large kidney section is shown with the outline of the scanned regions at 1.3µm and 6µm overlayed in green and blue respectively, there is no visible change in tissue morphology across these borders. Furthermore, immunohistochemistry (IHC) was performed on a Control and COVID lung biopsy sample (**Extended Data 7**) indicating that neither the tissue pre-processing nor the X-ray dose (lower for biopsy samples) appear to adversely affect the expected staining pattern for a panel of commonly used IHC markers. Whilst we have not yet performed IHC on highest dose areas (1.3 µm within a HiP-CT scanned organ, these data strongly suggest that HiP-CT is compatible with IHC). The only cases that led to visible tissue damages occurred during the first period of development of HiP-CT when the acquisition system stopped for several hours.

Supplementary Table 1: Statistics for COVID-19 lung microstructure analysis

Distance to Tissue - Multiple Comparison of Means - Tukey HSD, FWER=0.05			
**Group1**	**Group2**	**Mean_diff**	**p-adj**
**COVID$_C$**	**COVID$_S$**	-16.2224	0.001
**COVID$_C$**	**Control**	-21.638	0.001
**COVID$_S$**	**Control**	-5.4156	0.016
**F**	87.53	**P**	$5.36x10^{-9}$
Airspace connectivity - Multiple Comparison of Means - Tukey HSD, FWER=0.05			
**Group1**	**Group2**	**Mean_diff**	**p-adj**
**COVID$_C$**	**COVID$_S$**	-0.0	0.001
**COVID$_C$**	**Control**	-0.0	0.0337
**COVID$_S$**	**Control**	0.0	0.0805
**F**	13.26	**P**	0.00048
Airspace surface area to volume ratio - Multiple Comparison of Means - Tukey HSD, FWER=0.05			
**Group1**	**Group2**	**Mean_diff**	**p-adj**
**COVID$_C$**	**COVID$_S$**	0.3701	0.001
**COVID$_C$**	**Control**	0.6217	0.001
**COVID$_S$**	**Control**	0.2516	0.001
**F**	275.9	**P**	$1.48x10^{-12}$
Tissue thickness - Multiple Comparison of Means - Tukey HSD, FWER=0.05			
**Group1**	**Group2**	**Mean_diff**	**p-adj**
**COVID$_C$**	**COVID$_S$**	-718.5333	0.001
**COVID$_C$**	**Control**	-1029.573	0.001
**COVID$_S$**	**Control**	-311.0397	0.001
**F**	**128.1**	**P**	$3.72x10^{-10}$
Airway Diameter - Multiple Comparison of Means - Tukey HSD, FWER=0.05			
**Group1**	**Group2**	**Mean_diff**	**p-adj**
**COVID$_C$**	**COVID$_S$**	-229.8232	0.037
**COVID$_C$**	**Control**	207.59	0.062
**COVID$_S$**	**Control**	437.4132	0.001
**F**	**13.7**	**P**	0.0004

Supplementary Table 2: Voxel sizes, anatomical label, DOI, Energy, FOV and scan time for all samples in this work. All data can be accessed with image previews, videos and binned data versions available via the https://human-organ-atlas.esrf.eu search by volume of interest label. DOIs in this table link directly to the data download only.

Sample	Voxel size (µm)	Volume of Interest label	DOIs	Average detected (keV)	Field of view diameter (mm)	Scan time (hrs)
Donor 1 heart	25.08 µm	Complete organ	10.15151/ESRF-DC-572189991	~93 keV	95.2	18
	6.05 µm	Left and right ventricle muscle + ramus interventricularis anterior	10.15151/ESRF-DC-572190117	~95 keV	23	2
	2.22 µm	Left ventricle muscle	10.15151/ESRF-DC-572185639	~84 keV	8.4	1.6
Donor 1 left lung	25.08 µm	Complete organ	10.15151/ESRF-DC-572196058	~93 keV	145	24
	25.25 µm	FSC A&B	10.15151/ESRF-DC-572221247	~88 keV	145	1.3
	6.05 µm	VOI-06	10.15151/ESRF-DC-572232527	~89 keV	23	1.8
	6.5 µm	FSC A&B	10.15151/ESRF-DC-572235527	~88 keV	24.7	0.5
	2.45 µm	VOI-02	10.15151/ESRF-DC-572191782	~79 keV	9.3	1
	2.45 µm	VOI-06	10.15151/ESRF-DC-572194514	~79 keV	9.3	0.4
	2.51 µm	FSC A&B	10.15151/ESRF-DC-572216900	~77 keV	9.5	0.5
Donor 1 left kidney	25.08 µm	Complete organ	10.15151/ESRF-DC-572182553	~93 keV	85	2.5
	6.05 µm	Central column	10.15151/ESRF-DC-572183366	~89 keV	23	2
	1.29 µm	Central column	10.15151/ESRF-DC-572174012	~74 keV	4.9	1.2
Donor 1 spleen	25.08 µm	Complete organ	10.15151/ESRF-DC-572244468	~93 keV	85	2
	6.05 µm	Central column	10.15151/ESRF-DC-572244779	~89 keV	23	2
	1.29 µm	Central column	10.15151/ESRF-DC-572242562	~74 keV	4.9	2

Donor 2 brain	25.08 µm	Complete organ	10.15151/ESRF-DC-572252655	~93 keV	145	22
	6.05 µm	Cerebellum – occipital lobe	10.15151/ESRF-DC-572253460	~89 keV	24.7	1
	2.45 µm	Cerebellum	10.15151/ESRF-DC-572252431	~83 keV	9.3	1
Donor 2 kidney	25 µm	Complete organ	10.15151/ESRF-DC-572253769	~88 keV	85	6
	2.5 µm	Lateral transect	10.15151/ESRF-DC-572256027	~84 keV	9.5	1
Donor 3 upper lobe of left lung	26.38 µm	Complete upper lobe	10.15151/ESRF-DC-571998188	~74 keV	100.2	11
	25.25 µm	Complete upper lobe (bis)	10.15151/ESRF-DC-572101011	~79 keV	95.9	7.5
	6.5 µm	VOI-04-bis	10.15151/ESRF-DC-572118595	~78 keV	24.7	1.5
	6.24 µm	VOI-04	10.15151/ESRF-DC-572067336	~73 keV	23.7	1.7
	6.24 µm	VOI-03	10.15151/ESRF-DC-572062303	~73 keV	23.7	3.7
	2.22 µm	VOI-01.2b	10.15151/ESRF-DC-579010979	~81 keV	8.4	3.5
	2.22 µm	VOI-03.2	10.15151/ESRF-DC-572038314	~81 keV	8.4	3.7
	2.22 µm	VOI-08.2	10.15151/ESRF-DC-572099177	~81 keV	8.4	0.6
	2.2 µm	VOI-03b-bis	10.15151/ESRF-DC-572116463	~74 keV	8.4	0.8
	2.2 µm	VOI-aa-bis	10.15151/ESRF-DC-572118232	~74 keV	8.4	1.8
	0.65 µm	VOI-aa	10.15151/ESRF-DC-572172172	~83 keV	2.5	0.5
Donor 3 upper lobe of right lung	2.25 µm	Core biopsy	10.15151/ESRF-DC-578823778	~44 keV	8.6	1.8
Donor 4 lower lobe of right lung	2.25 µm	Core biopsy	10.15151/ESRF-DC-574369994	~82 keV	8.6	1
