# Supplementary material for: Imaging intact human organs with local resolution of cellular structures using hierarchical phase-contrast tomography
Source: Nat Methods. 2021 Nov 4;18(12):1532–41. doi: 10.1038/s41592-021-01317-x (PMC8648561; doi:10.1038/s41592-021-01317-x)
Supplement: Supplementary file 2 [file 41592_2021_1317_MOESM2_ESM.pdf]

nature research

Corresponding author(s): Peter D. Lee, D,D Jonigk, M. Ackermann

Last updated by author(s): 13/9/21

Reporting Summary

Nature Research wishes to improve the reproducibility of the work that we publish. This form provides structure for consistency and transparency in reporting. For further information on Nature Research policies, see our Editorial Policies and the Editorial Policy Checklist.

Statistics

For all statistical analyses, confirm that the following items are present in the figure legend, table legend, main text, or Methods section.

n/a	Confirmed	
☐	☒	The exact sample size (*n*) for each experimental group/condition, given as a discrete number and unit of measurement
☐	☒	A statement on whether measurements were taken from distinct samples or whether the same sample was measured repeatedly
☐	☒	The statistical test(s) used AND whether they are one- or two-sided<br>*Only common tests should be described solely by name; describe more complex techniques in the Methods section.*
☒	☐	A description of all covariates tested
☐	☒	A description of any assumptions or corrections, such as tests of normality and adjustment for multiple comparisons
☐	☒	A full description of the statistical parameters including central tendency (e.g. means) or other basic estimates (e.g. regression coefficient) AND variation (e.g. standard deviation) or associated estimates of uncertainty (e.g. confidence intervals)
☐	☒	For null hypothesis testing, the test statistic (e.g. F, t, r) with confidence intervals, effect sizes, degrees of freedom and P value noted<br>*Give P values as exact values whenever suitable.*
☒	☐	For Bayesian analysis, information on the choice of priors and Markov chain Monte Carlo settings
☒	☐	For hierarchical and complex designs, identification of the appropriate level for tests and full reporting of outcomes
☐	☒	Estimates of effect sizes (e.g. Cohen's *d*, Pearson's *r*), indicating how they were calculated

Our web collection on statistics for biologists contains articles on many of the points above.

Software and code

Policy information about availability of computer code

Data collection	HiP-CT data was acquired on ESRF BM05
Data analysis	HiP-CT data were reconstructed using custom code written in Matlab2017 available at: https://github.com/HiPCTProject/Tomo_Recon and the PyHST2 software package (https://software.pan-data.eu/software/74/pyhst2)<br>Volume renderings were performed using 3D software packages, notably VGStudioMax 3.2.4 (Volume Graphics, Heidelberg, Germany), Amira v2019.6 and Dragonfly 2020.2.0<br>Image quality comparison was performed using Matlab 2020b (ssim function). ImageJ v2.1.0/1.53c Java 1.8.0_66 and pecific plugins - BoneJ v7.0.11 and MorpholibJ v1.4.2.1 were used for lung morphology analysis, pyradiomicsv3.0 (https://pyradiomics.readthedocs.io/en/latest/index.html) was used to calculate skew and kurtosis of image histograms. FSC analysis code was slightly modified from https://github.com/bionanoimaging/cellSTORM-MATLAB/blob/master/Functions/FSC.m

For manuscripts utilizing custom algorithms or software that are central to the research but not yet described in published literature, software must be made available to editors and reviewers. We strongly encourage code deposition in a community repository (e.g. GitHub). See the Nature Research guidelines for submitting code & software for further information.

Data

Policy information about availability of data

All manuscripts must include a data availability statement. This statement should provide the following information, where applicable:

- Accession codes, unique identifiers, or web links for publicly available datasets
- A list of figures that have associated raw data
- A description of any restrictions on data availability

Image data that support the findings of this study are publicly available (with sign in) from the ESRF data repository. human-organ-atlas.esrf.eu
Individual DOIs for all datasets are listed in Supplementary Table 2

Field-specific reporting

Please select the one below that is the best fit for your research. If you are not sure, read the appropriate sections before making your selection.

☒ Life sciences ☐ Behavioural & social sciences ☐ Ecological, evolutionary & environmental sciences

For a reference copy of the document with all sections, see nature.com/documents/nr-reporting-summary-flat.pdf

Life sciences study design

All studies must disclose on these points even when the disclosure is negative.

Sample size	Number of samples was determined by the beamtime available.<br>Fig 1., N= 2 high resolution sub-regions from 1 lung were compared. From these sub-regoins N = 200 image slices were used for structural similarity index comparison,<br>FSC estimation: N =9 sub-volumes from two independently reconstructed image volumes were analyzed by FSC for resolution estimation.<br>Figure 2 - N= 6 Organs were scanned<br>Figure 3. N=1 Kidney was analyzed hierarchically, N=13 glomeruli were analyzed for morphology<br>Fig 4 constructed sub-volumes used N= 6 per group<br>N = 4 Purkinje cells were segmented and CNR ratio calculated<br>N =9 (3 per image) regions were analyzed for SNR ratio in lateral kidney transect data
Data exclusions	For control organs - two autopsies were performed the organ which appeared the healthiest from a gross pathological point of view was selected for this study.
Replication	The COVID-lung and Control brain have been rescanned since the initial scans (data presented in the manuscript). Whilst there has been a small amount of overall organ shrikage over this time (~4months) there are no tissue specific changes which affect the results or conclusions of this work. The second Kidney from Donor 1 was also scanned with similar results (included in Fig. 1)
Randomization	This study involved anatomical analysis intact human organs. In most analyses, fundamental organizational of tissue morphology was examined, rather than comparing experimental and control samples. Therefore, randomization was not necessary.
Blinding	Blinding was not performed between control and COVID lungs.

Reporting for specific materials, systems and methods

We require information from authors about some types of materials, experimental systems and methods used in many studies. Here, indicate whether each material, system or method listed is relevant to your study. If you are not sure if a list item applies to your research, read the appropriate section before selecting a response.

Materials & experimental systems

n/a	Involved in the study	
☐	☒	Antibodies
☒	☐	Eukaryotic cell lines
☒	☐	Palaeontology and archaeology
☒	☐	Animals and other organisms
☐	☒	Human research participants
☒	☐	Clinical data
☒	☐	Dual use research of concern

Methods

n/a	Involved in the study	
☒	☐	ChIP-seq
☒	☐	Flow cytometry
☒	☐	MRI-based neuroimaging

Antibodies

Antibodies used	CD31, TTF-1 RTU and Fibrin
Validation	CD31 1:75 (M0823, Agilent Dako, California, USA), TTF-1 RTU (790-4756, Hoffmann-La Roche, Basel, Swiss) and Fibrin 1:500 (MABS2155, Merck Millipore , Massachusetts, USA) were stained on a VENTANA BenchMark ULTRA (Hoffmann-La Roche, Basel, Swiss) with the aforementioned dilutions and pretreatment and incubation times according to the manufacturers advice.

Human research participants

Policy information about studies involving human research participants

Population characteristics	Donor 1, from which the heart, lung, kidney, and spleen were imaged, was a 94-year-old, 45kg, 140cm female with right sylvian and right cerebellar stroke, cognitive disorders of vascular origin, depressive syndrome, atrial fibrillation and hypertensive heart disease, micro-crystalline arthritis (gout), right lung pneumopathy (3 years before death), cataract of the left eye, squamous cell carcinoma of the skin (left temporal region).<br>Donor 2, from which the brain was imaged, was a 69-year-old, 40 kg, 145 cm female, with type 2 diabetes, pelvic radiation to treat cancer of the uterus, right colectomy (benign lesion on histopathology), bilateral nephrostomy for acute obstructive renal failure, cystectomy, omentectomy and peritoneal carcinoma with occlusive syndrome.<br>Donor 3, The entire upper left lobe and a core biopsy from the periphery of the right upper lobe were obtained from a 54-year-old male patient who died from COVID-19, 21 days after hospitalisation. Treatment involved mechanical ventilation. Regarding comorbidities, arterial hypertension and type II diabetes were not diagnosed prior to death.<br>Donor 4, a biopsy from the right lung, lower lobe. Patient was a male 77 years, resection of the segment due to small pulmonary adenocarcinoma (1.4 cm) coronary heart disease, arterial hypertension, chronic rheumatic disease (polymyalgia rheumatica).
Recruitment	Control organs were donated to the LADAF for scientific training and research purposes.<br>COVID-19 donors were recruited from the Patients who died from COVID-19-infection, grouped into early, mid and late stage acute respiratory distress syndrome. Patient age group to average 70, including controls and were collected by the Hannover Unified Biobank at Medizinische Hochschule, Hannover.
Ethics oversight	Hannover Institute of Pathology at Medizinische Hochschule, Hannover (Ethics vote no. 9022_BO_K_2020). Laboratoire d'Anatomie des Alpes Françaises (LADAF), Health Research Authority and Integrated Research Application System (HRA & IRAS) (200429)

Note that full information on the approval of the study protocol must also be provided in the manuscript.
